# Hypermethylation of ZNF154 promotes malignant potential of ovarian cancer cells by diminishing ZNF154/KAP1-mediated ROMO1 repression

**DOI:** 10.1038/s41419-026-08823-w

**Published:** 2026-05-02

**Authors:** Mingbiao Wei, Yuxia Xu, Ling Deng, Wei Wei, Dongyi Ling, Shanshan Zhen, Ran Zhou, Wenjian Cen, Xu Zhang, Mayan Huang, Jundong Li, Shumei Yan, Qin Li, Ziming Du

**Affiliations:** 1 State Key Laboratory of Oncology in South China, Guangdong Provincial Clinical Research Center for Cancer, Sun Yat-sen University Cancer Center, Guangzhou, PR China; 2 Department of Molecular Diagnostics, Sun Yat-sen University Cancer Center, Guangzhou, PR China; 3 Department of Gynecological Oncology, Sun Yat-sen University Cancer Center, Guangzhou, PR China; 4 Department of Intensive Care, Sun Yat-sen University Cancer Center, Guangzhou, PR China; 5 Department of Pathology, Sun Yat-sen University Cancer Center, Guangzhou, PR China; 6 Guangdong Provincial Key Laboratory of Malignant Tumor Epigenetics and Gene Regulation, Guangdong-Hong Kong Joint Laboratory for RNA Medicine, Sun Yat-sen Memorial Hospital, Guangzhou, PR China; 7 Medical Research Center, Sun Yat-sen Memorial Hospital, Guangzhou, PR China

**Keywords:** Tumour-suppressor proteins, Prognostic markers

## Abstract

Accumulating evidence demonstrates that the silencing of tumor suppressor genes by aberrant DNA methylation contributes to the initiation and progression of ovarian cancer (OC), while the systemic methylation profile and the key driver methylation events need to be further explored. Here, by analysing public databases and our resources, we identified the hypermethylation of *ZNF154* promoter as a key driver of OC malignancy, which was mediated by the DNA methyltransferase complex DNMT1/UHRF1. Using CRISPR/dCas9-TET1CD, a tool for targeted demethylation, we successfully decreased the methylation level of *ZNF154* promoter and reactivated ZNF154 expression, which in turn inhibited the proliferation, migration, and invasion of OC cells. Mechanistically, ZNF154 interacted with KAP1 and directly bound to the *ROMO1* promoter, transcriptionally repressing ROMO1 expression, thereby reducing MMP2 and phosphorylated ERK to impede OC progression. Clinically, *ZNF154* hypermethylation was correlated with its reduced expression and poor prognosis in OC patients. These findings underscore a pivotal role of aberrant *ZNF154* methylation in OC pathogenesis and highlight its potential as both a therapeutic target and a prognostic biomarker for OC patients.

## Introduction

Ovarian cancer (OC) is one of the most common cancers and a primary contributor to women’s gynecologic cancer-related mortality, with over 300,000 new cases and 207,252 deaths annually [1, 2]. Owing to nonspecific early symptoms and inadequate screening methods, ~80% of OC cases are discovered at advanced stages, severely limiting treatment options and resulting in a poor 5-year survival rate [3]. Therefore, a deeper understanding of the mechanisms driving OC initiation and progression is essential for identifying novel therapeutic targets as well as diagnostic markers.

Epigenetic changes, which involve modifications without changing the sequence of DNA, play pivotal roles in cellular adaptation to internal and external environmental stimuli [4–6]. DNA methylation, the most well-researched epigenetic modification, refers to the addition of a methyl group to the cytosine ring in cytosine-phosphate-guanine (CpG) sites. This process is essential for maintaining genomic stability and modulating gene expression [7, 8]. Accumulating evidence indicates that aberrant DNA methylation drives the initiation and progression of OC [9, 10]. Notably, hypomethylation activates oncogenes, whereas hypermethylation silences tumor suppressor genes (TSGs) [9]. underscoring the need for further investigation into DNA methylation dysregulation in OC.

Zinc finger proteins (ZFPs), which constitute the largest family of transcription factors in mammals, bind to promoter regions of target genes through their zinc finger domains to regulate gene expression [11–13]. Alterations in ZFPs expression, frequently controlled epigenetically via DNA methylation, contribute to tumorigenesis. For instance, ZNF382 is hypermethylated in esophageal cancer and functions as a tumor suppressor by inhibiting the Wnt/β-catenin pathway [14]. Similarly, ZNF471 is frequently silenced by promoter methylation in renal cell carcinoma, esophageal cancer, and gastric cancer. Downregulation of ZNF471 promotes tumorigenesis through multiple pathways [15–17]. Additionally, reduced expression of ZBTB16 via promoter hypermethylation promotes the proliferation and invasion of lung adenocarcinoma cells [12]. Moreover, abnormal hypermethylation contributes to downregulation of ZNF334, thereby promoting progression in triple-negative breast carcinoma and colorectal cancer [18, 19].

Zinc finger protein 154 (ZNF154) is a novel ZFP belonging to the Krüppel-associated box zinc finger proteins (KRAB-ZFPs) family, and its gene is located on 19q13.43 [20]. Previous studies have reported that *ZNF154* promoter hypermethylation is a common epigenetic event in various malignant tumors, including gastric cancer, invasive breast carcinoma, and endometrial cancer [21–24]. This epigenetic alteration shows potential prognostic or diagnostic value. In OC, multiple studies have also confirmed the presence of *ZNF154* promoter hypermethylation, which either alone or in combination with other markers, may hold preliminary potential in the diagnosis and assessment of OC [23–27]. Notably, ZNF154 has been reported to function as a tumor suppressor and inhibit the proliferation and migration of esophageal carcinoma cells [28]. However, its functional role and underlying mechanisms in OC remain poorly understood. This work focused on determining the methylation profile and transcriptional activity of *ZNF154* in OC, assessing its clinical relevance to patient outcomes. Additionally, we characterized the biological contributions and underlying molecular mechanisms through which ZNF154 influences OC pathogenesis.

## Materials and methods

### Public datasets and bioinformatic analysis

Several public datasets, including The Cancer Genome Atlas (TCGA), Genotype-Tissue Expression (GTEx), Gene Expression Omnibus (GEO), KM Plotter database and UALCAN database were used in the present study. Specifically, DNA methylation data of OC tissues were downloaded from the datasets of GSE146552 and TCGA. Gene expression data were obtained from the datasets of TCGA, GTEx, GSE137238, GSE146553, UALCAN and KM Plotter databases. To identify the differentially hypermethylated CpG sites in OC tissues, we analyzed 27 K methylation array data from TCGA, which were subsequently validated using 450 K methylation array data from GSE146552. Based on the methylation profile, we examined the expression of ZNF154 across TCGA, GTEx and GSE137238. Additionally, the relationship between gene expression and DNA methylation was evaluated using Pearson correlation analysis.

### Patient samples

Two cohorts were included in the present study. Cohort I comprised formalin-fixed, paraffin-embedded (FFPE) tissues, consisting of 122 OC tissues and 30 paired normal fallopian tube tissues. Cohort II consisted of serum samples from 29 healthy controls and 48 OC patients. All patient samples were obtained from Sun Yat-sen University Cancer Center (SYSUCC). The serum samples were used exclusively for MethyLight assay, while the FFPE tissues were used for both MethyLight assay and immunohistochemistry (IHC) staining. Detailed clinicopathological information for all samples is presented in Supplementary Tables S1, S2.

### DNA extraction and Bisulfite modification

QIAamp® DNA FFPE Tissue kit (Qiagen) and QIAamp DNA Blood Mini Kit (Qiagen) were used to extract genomic DNA and cell-free DNA (cfDNA), respectively. The EZ DNA Methylation Kit (Zymo Research) was used to bisulfite modify extracted DNA.

### MethyLight assay

MethyLight assay was performed to detect the methylation level of the *ZNF154* promoter in both tissue and serum samples. The PCR primers and TaqMan probe were specifically designed using Oligo 7 software to amplify bisulfite-modified DNA sequences of *ZNF154*. *ALU* was used as an endogenous reference gene for normalizing the input DNA. The probe and primers for *ALU* were generated based on previously published studies. Supplementary Table S3 contains a list of all probes and primers utilized in the MethyLight assay.

MethyLight assay was conducted on 7500 Real-time PCR system (ABI) in duplicate, using a 25 µL reaction volume. Each reaction contained 3 µL of bisulfite-modified DNA, 0.6 µM primer, 0.3 µM probe, 12.5 µL of 2× Methylation PCR Master Mix. The amplification conditions were as follows: a denaturation at 95 °C for 20 min, followed by 45 cycles of 95 °C for 20 s (denaturation) and 56 °C for 1 min (annealing/extension). M.SssI methyltransferase (New England Biolabs) was used to methylate the DNA extracted from normal fallopian tube tissue, which served as the positive control.

### IHC stainings

IHC staining for ZNF154 (HPA076284, Sigma-Aldrich) was conducted on 4 μm slices of both normal fallopian tube tissues and OC tissues. Two independent, blinded pathologists evaluated the IHC scores, which ranged from 0 to 12. The percentage of immunopositive cells (0%, 0; 1–25%, 1; 26–50%, 2; 51–75%, 3; >75%, 4) and the degree of positive staining (negative, 0; weak, 1; moderate, 2; strong, 3) were multiplied to determine the score. ZNF154 expression was categorized as either low (IHC score <3) or high (IHC score ≥3).

### Cell culture

Human OC cell lines A2780, SKOV3 and human embryonic kidney cell line HEK 293T were cultured in DMEM (Gibco) supplemented with 10% fetal bovine serum (FBS). All cell lines were grown in 5% CO_2_ humidified atmosphere at 37 °C. Short tandem repeats (STR) profiling was used to identify the cell lines.

### Plasmid construction

The human *Flag-ZNF154* gene was cloned into pLV-EF1a-IRES-Puro vector, and the human *ROMO1* gene was cloned into pLV-CMV-PGK-Bla vector. The pGL4 basic vector was modified to include the *ROMO1* promoter region. The pLKO.1-puro vector was modified to include the shRNAs that target *KAP1* in order to knock down endogenous KAP1. To achieve targeted demethylation, the sgRNAs that target *ZNF154* were cloned into the CRISPR/dCas9-TET1CD vector. Supplementary Table S4 lists the sequences of the indicated sgRNAs and shRNAs.

### Cell viability assay

The CCK-8 assay was performed using CCK-8 reagent (DOJINDO) in accordance with the manufacturer’s instructions to assess proliferation ability.

### Colony formation assay

A total of 800 cells were plated into 6-well culture dishes and maintained for 10–14 days. Following the incubation period, cellular colonies were fixed in absolute methanol and stained with 0.1% crystal violet solution. ImageJ software was used to count the colonies.

### Migration and invasion assays

The migration and invasive capabilities of OC cells were evaluated by transwell assays using 8.0 μm transwell chambers (Falcon, BD Bioscience), with or without Matrigel coating (medium: Matrigel ratio = 15:1). For migration assay, A2780 (8 × 10^4^) or SKOV3 (4 × 10^4^) cells in 200 µL medium without serum were plated into the upper chamber. The lower chamber (24-well plate) received 700 µL medium containing 10% FBS. Cells on the lower membrane surface were fixed in absolute methanol and stained with 0.1% crystal violet solution following a 24-h incubation period. For invasion assay, upper chambers were pre-coated with Matrigel prior to cell seeding.

### RNA isolation and quantitative real-time PCR (qRT-PCR)

TRIzol reagent (15596026CN, Invitrogen) was used to extract RNA from the indicated cells. The PrimeScriptTM RT reagent Kit (RR047A, Takara) was then used to create cDNA. SYBR®Green Master Mix (QPK-201, TOYOBO) was used for qRT-PCR analyses. Supplementary Table S5 lists the precise primer sequences used for qRT-PCR.

### Western blot

Western blotting was carried out according to the accepted procedure using the following antibodies: anti-ROMO1 (MG615612S, Abmart), anti-Flag (F1804, Sigma-Aldrich), anti-Myc (60003-2-Ig, Proteintech), anti-KAP1 (66630-1-Ig, Proteintech), anti-MMP2 (T57164S, Abmart), anti-ERK1/2 (T40071S, Abmart), anti-Phospho-Erk1 (T202/Y204) + Erk2 (T185/Y187) (T40072S, Abmart), anti-GAPDH (60004-1-Ig, Proteintech).

### Cleavage under targets and tagmentation (CUT&Tag) assay

CUT&Tag assays were conducted following established protocols [29]. Primary antibodies employed included: anti-Flag (F1804, Sigma-Aldrich) and IgG (12-371, Millipore).

### Chromatin immunoprecipitation (ChIP) and ChIP-qPCR Assays

As directed by the manufacturer, the SimpleChIP® Plus Sonication Chromatin IP Kit (56383S, Cell Signaling Technology) was used to perform the ChIP assay. In summary, formaldehyde was used to fix 4 × 10^6^ cells, which were then lysed, sonicated, immunoprecipitated, and their DNA purified. Anti-Flag (F1804, Sigma-Aldrich) and IgG (sc-2025, Santa Cruz) were the antibodies utilized for immunoprecipitation. The purified DNA was analyzed by qRT-PCR using SYBR®Green Master Mix (QPK-201, TOYOBO) and special primers (ROMO1-ChIP-F: TTCGACCGTGTCAAAATGGG, ROMO1-ChIP-R: GCCGGAAGGTCTGGTTGTC).

### Luciferase reporter assay

Human *ROMO1* promoter and its mutant variants were cloned into the pGL4 basic vector. 2 × 10⁵ A2780 cells were seeded in 12-well plates and transfected with the following plasmids: promoter-luciferase plasmid, pRL-TK Renilla reporter plasmid, and pLV-ZNF154 or its control vector. After 48 h, cells were lysed and subjected to dual-luciferase assays using a commercial kit (E1910, Promega) according to the manufacturer’s protocol.

### Coimmunoprecipitation (coIP)

Cell lysis was performed on ice for 30 min using RIPA buffer containing protease/phosphatase inhibitor cocktails. After centrifugation at 12,000 × *g* (4 °C, 15 min), the clarified supernatant was subjected to overnight immunoprecipitation at 4 °C with 2 μg of specific antibodies: anti-Flag (F1804, Sigma), anti-Myc (16286-1-AP, Proteintech), anti-KAP1 (66630-1-Ig, Proteintech), or control IgG (sc-2025, Santa Cruz). Protein G magnetic beads (10004D, Invitrogen) were subsequently added for 4 h at 4 °C. Following four washes with lysis buffer, bound proteins were eluted in SDS loading buffer for immunoblotting.

### Animal experiments

Female immunodeficient mice (4-week-old BALB/c nude and NSG strains [NOD.Cg-Prkdc<scid>Il2rg<tm1Wjl>/SzJ]) were procured from GemPharmatech and SYSUCC (both in Guangdong, China), respectively. Animals were randomly assigned to five groups using a computer-generated sequence and were subsequently analyzed by an investigator blinded to the group allocation. To generate subcutaneous xenografts, 1 × 10⁶ tumor cells suspended in PBS were injected into the right scapular region of nude mice. Following a 5-week observation period, mice were euthanized for tumor excision and gravimetric analysis. For metastatic modeling, 5 × 10⁶ cells were administered intraperitoneally to NSG mice. After 4 weeks, macroscopic peritoneal metastatic nodules were counted and their mass quantified. Animal cohort size was determined by the 3R principle of Reduction. All procedures involving experimental animals strictly adhered to the Guide for the Care and Use of Laboratory Animals.

### Statistical analysis

Statistical analyses were conducted with R (v4.1.3), SPSS Statistics (v23), and GraphPad Prism 9. For these analyses, data from all available clinical samples and animal specimens were included. Overall survival curves were generated by the Kaplan–Meier method, with between-group comparisons assessed via log-rank testing. Categorical variables were evaluated using χ² or Fisher’s exact tests as appropriate. Continuous variables were analyzed using the appropriate statistical tests (Student’s *t* test, Welch’s *t* test, Mann–Whitney *U* test, ANOVA, Wilcoxon test, or Kruskal–Wallis *H* test) based on whether the data met assumptions of normality and homogeneity of variance. A two-sided *P*-value threshold of 0.05 defined statistical significance.

## Results

### *ZNF154* promoter hypermethylation correlates with poor prognosis in OC

**Fig. 1:**
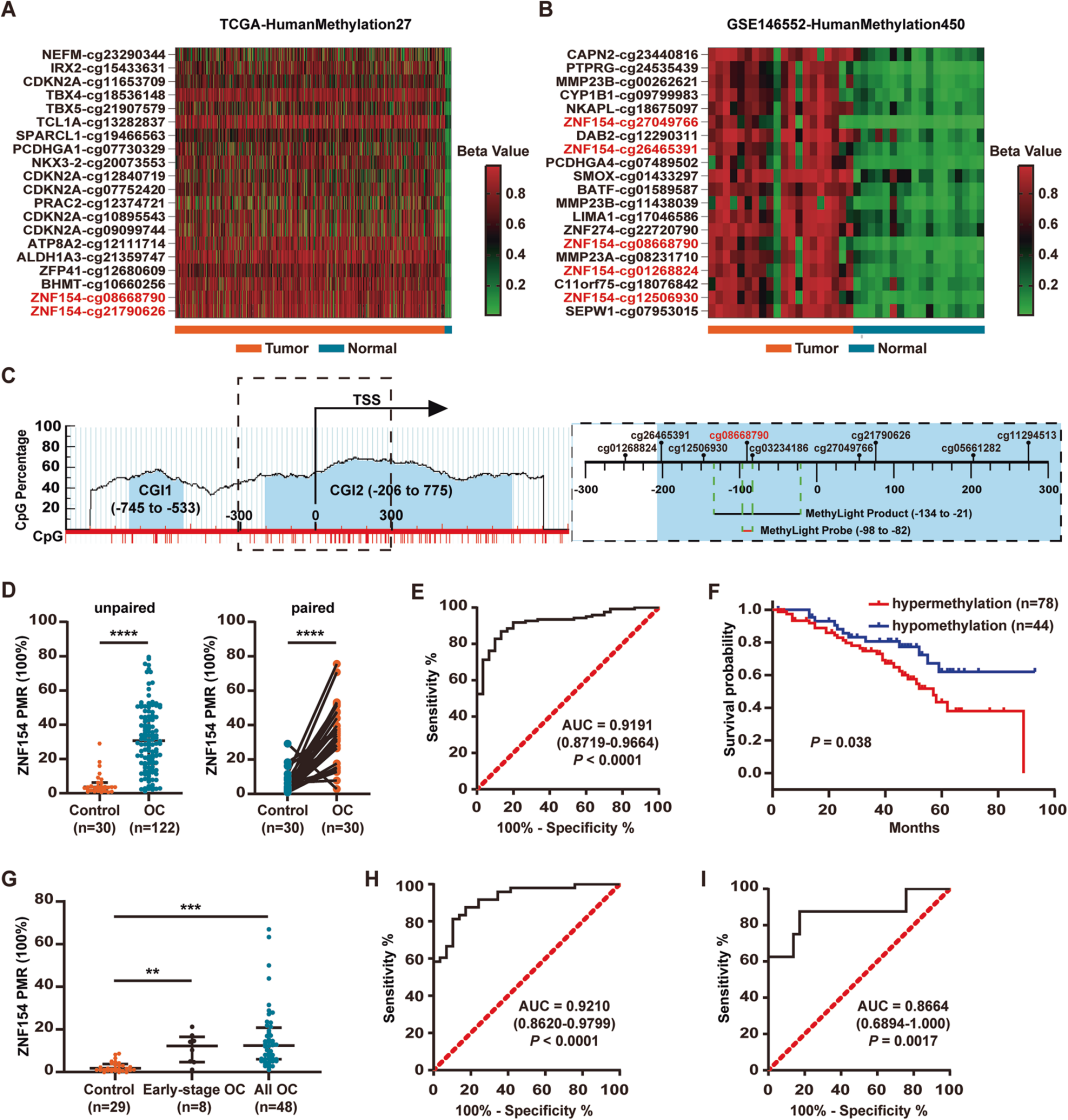
*ZNF154* promoter hypermethylation correlates with poor prognosis in OC. **A** Heatmaps showing 20 representative differentially hypermethylated CpG sites in OC tissues compared to normal tissues, based on The Cancer Genome Atlas (TCGA) data (OC, *n* = 582; normal, *n* = 12). **B** Heatmaps showing 20 representative differentially hypermethylated CpG sites in OC tissues compared to normal tissues, based on Gene Expression Omnibus (GEO) data (OC, *n* = 20; normal, *n* = 18). **C** Schematic illustration depicting the location of CpG islands in the promoter region of *ZNF154*. CpG sites (vertical bars) from −300 bp to +300 bp relative to the TSS are shown, along with the location of the probe used for the MethyLight assay. **D** PMR in unpaired (left) and paired (right) comparisons of OC tissues and normal fallopian tube tissues. **E** ROC curve demonstrating the ability of PMR to differentiate OC tissues from normal fallopian tube tissues (OC, *n* = 122; normal, *n* = 30). **F** Kaplan–Meier survival analysis based on PMR levels in OC patients (*n* = 122). **G** PMR in serum samples from all-stage and early-stage (I–II) OC patients and healthy controls. **H** ROC curve depicting the ability of PMR to distinguish OC patients from healthy controls based on serum samples (OC, *n* = 48; healthy controls, *n* = 29). **I** ROC curve depicting the ability of PMR to discriminate early-stage OC patients from healthy controls based on serum samples (early-stage OC, *n* = 8; healthy controls, *n* = 29). Data are presented as median (interquartile range). The Wilcoxon signed-rank test, Mann–Whitney *U* test, and Kruskal–Wallis *H* test were used for statistical comparisons as appropriate. **** *p* < 0.0001. CGI CpG island, TSS transcription start site, OC ovarian cancer, PMR percent methylated reference, ROC receiver operating characteristic, AUC area under the curve.

To identify DNA methylation-driven genes in OC, we analyzed TCGA methylation profiles from OC patients. A heatmap displayed the top 20 differentially hypermethylated CpG sites (Fig. 1A). Strikingly, cg21790626 and cg08668790, both located in the promoter region of *ZNF154*, ranked among the most differentially hypermethylated sites in OC tissues (Fig. 1A). This finding was further validated using data from the GEO database (Fig. 1B). Integrated analysis of TCGA and GEO data established *ZNF154* as a potential DNA methylation-driven gene in OC pathogenesis. Using the MethPrimer tool, we identified two CpG islands (CGIs) near the transcriptional start site (TSS) of *ZNF154* (Fig. 1C). Based on consistent hypermethylation of the cg08668790 site in OC tissues across TCGA and GEO datasets, we established a MethyLight assay to measure the percent of methylated reference (PMR) at this site in Cohort I, which included 122 OC tissue samples and 30 paired normal fallopian tube tissues. OC samples exhibited significantly elevated PMR compared to normal controls (Fig. 1D). Receiver operating characteristic (ROC) curve analysis demonstrated the diagnostic potential of *ZNF154* PMR for OC, achieving 86.89% sensitivity, 86.67% specificity, and an AUC of 0.92 (95% CI: 0.87–0.97) with an optimal threshold of 9.6% PMR (Fig. 1E). Furthermore, patients exhibiting *ZNF154* hypermethylation showed significantly reduced overall survival compared to those with hypomethylation (Fig. 1F).

Given growing evidence that aberrant promoter methylation in cfDNA provides a non-invasive approach for cancer detection, we also assessed the clinical diagnostic value of *ZNF154* PMR in serum samples from OC patients. Cohort II serum specimens (48 OC cases, 29 healthy controls) were analyzed by MethyLight assay. As anticipated, *ZNF154* PMR was significantly elevated in both all OC patients and early-stage (I–II) patients compared with controls (Fig. 1G). ROC analysis revealed considerable discriminative capacity for OC diagnosis (AUC = 0.92; 95% CI: 0.86–0.98), with 81.25% sensitivity and 89.66% specificity at the optimal threshold (Fig. 1H). For these early-stage cases, the ROC curve further indicated moderate diagnostic efficacy of serum *ZNF154* PMR with 87.50% sensitivity and 82.76% specificity (Fig. 1I).

### ZNF154 downregulation is associated with hypermethylation and predicts poor prognosis in OC

**Fig. 2:**
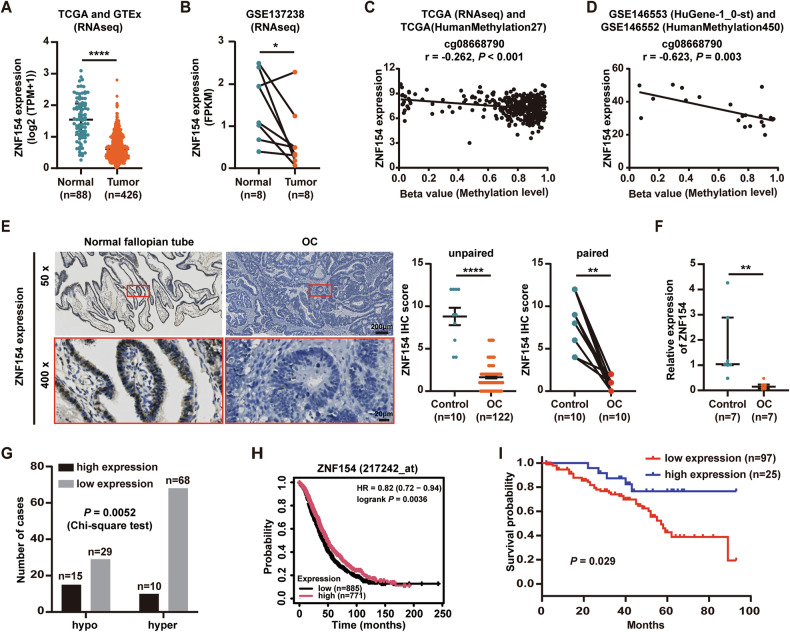
ZNF154 downregulation is associated with hypermethylation and predicts poor prognosis in OC. **A** *ZNF154* mRNA expression in OC tissues compared to normal tissues based on a combined dataset from TCGA and Genotype-Tissue Expression (GTEx). **B** *ZNF154* mRNA expression in OC tissues compared to normal tissues in the GEO dataset. **C**, **D** Pearson correlation analysis of the relationship between the methylation level of a specific CpG site (cg08668790) and *ZNF154* gene expression in the TCGA (*n* = 369) and GEO (*n* = 20) datasets. **E** Immunohistochemical analysis of ZNF154 protein expression in OC and normal fallopian tube tissues. The left and right panels display representative images and statistical plots, respectively. **F** qRT-PCR analysis of *ZNF154* mRNA expression in OC and normal fallopian tube tissues. **G** Correlation analysis between ZNF154 protein expression and PMR in OC tissues (χ² test). **H** Kaplan–Meier survival analysis based on *ZNF154* mRNA expression in OC patients (*n* = 1656) using the Kaplan–Meier Plotter tool. **I** Kaplan–Meier survival analysis based on ZNF154 protein expression in OC patients (*n* = 122). Data are presented as median (interquartile range). The Wilcoxon signed-rank test and Mann–Whitney *U* test were used for statistical comparisons as appropriate. *p < 0.05; ****p < 0.0001. hypo hypomethylation, hyper hypermethylation.

The above findings suggested that hypermethylation of *ZNF154* might be essential for the development of OC by downregulating ZNF154 expression. To further investigate this, we analyzed ZNF154 expression in TCGA, GTEx, and GEO databases. As anticipated, *ZNF154* mRNA expression was considerably lower in OC tissues than in normal tissues (Fig. 2A, B). Moreover, there was a negative correlation between *ZNF154* mRNA expression and the methylation level at the cg08668790 site (Fig. 2C, D). Immunohistochemistry and qRT-PCR analyses of clinical specimens further confirmed diminished ZNF154 expression in OC versus normal fallopian tube tissues (Fig. 2E, F). And the protein loss was significantly associated with promoter hypermethylation (Fig. 2G). Survival analysis revealed that OC patients with higher expression levels of ZNF154 showed better prognosis in both Kaplan–Meier Plotter (KMplot) web-based tool and our patient cohort (Fig. 2H, I). Taken together, our findings suggest that hypermethylation of *ZNF154* leads to its downregulation, which is also a predictor of poor prognosis in OC.

### ZNF154 is silenced by DNMT1/UHRF1-mediated methylation and functions as a tumor suppressor in OC

**Fig. 3:**
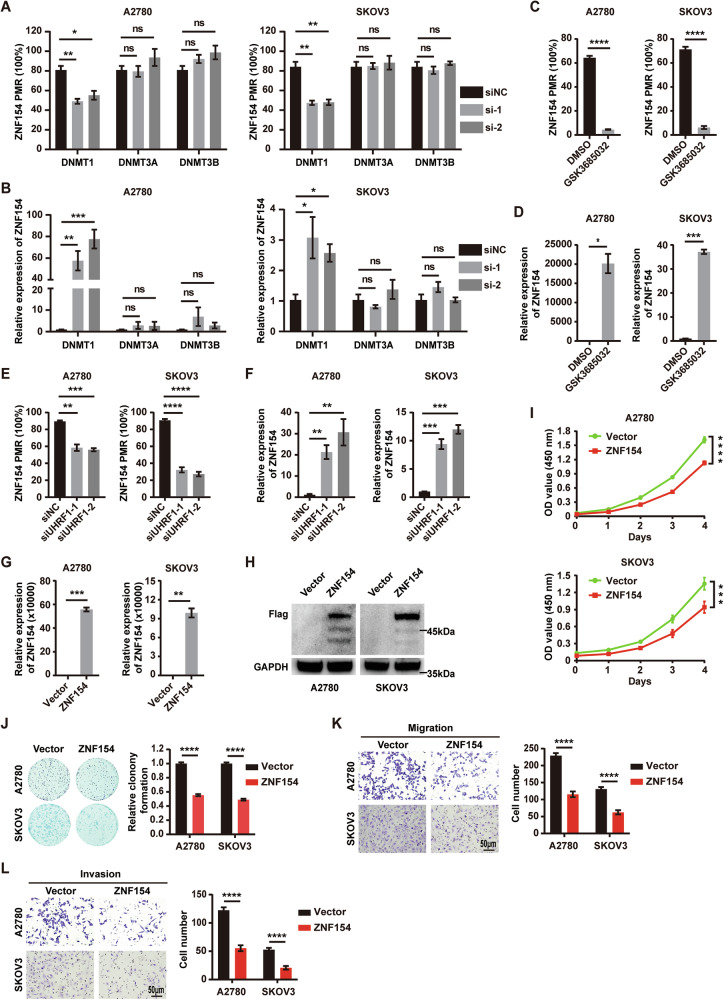
ZNF154 is silenced by DNMT1/UHRF1-mediated methylation and functions as a tumor suppressor in OC. **A** MethyLight assay results showing that knockdown of DNMT1, but not DNMT3A or DNMT3B, reduced the methylation level of *ZNF154*. **B** qRT-PCR analysis showing that knockdown of DNMT1, but not DNMT3A or DNMT3B, increased the expression of *ZNF154*. **C** *ZNF154* PMR in A2780 and SKOV3 cells treated with GSK3685032 (5 μM) for 4 days. **D** *ZNF154* mRNA levels in A2780 and SKOV3 cells treated with GSK3685032 (5 μM) for 4 days. **E** *ZNF154* PMR in A2780 and SKOV3 cells with or without UHRF1 knockdown. **F** *ZNF154* mRNA levels in A2780 and SKOV3 cells with or without UHRF1 knockdown. **G** *ZNF154* mRNA expression in A2780 and SKOV3 cells with or without ZNF154 overexpression. **H** Flag-ZNF154 protein expression in A2780 and SKOV3 cells with or without ZNF154 overexpression. **I** CCK-8 assay in cells with or without ZNF154 overexpression. **J** Colony formation assay in cells with or without ZNF154 overexpression (left panel), with accompanying quantitative analysis (right panel). **K** Transwell migration assay of cells with or without ZNF154 overexpression (left panel) and the accompanying quantitative analysis (right panel). **L** Transwell invasion assay of cells with or without ZNF154 overexpression (left panel) and the accompanying quantitative analysis (right panel). Data are presented as mean ± SEM. The Student’s *t* test, Welch’s *t* test, one-way ANOVA and two-way ANOVA were used for statistical comparisons as appropriate. *p < 0.05; **p < 0.01; ***p < 0.001; ****p < 0.0001; ns not significant.

DNA methylation is established and maintained through DNA methyltransferases (DNMTs) [30–32]. Humans have five different DNMTs: DNMT1, DNMT2, DNMT3A, DNMT3B, and DNMT3L. Among these, only DNMT1, DNMT3A and DNMT3B catalyse the addition of methyl groups to genomic DNA [4]. To identify the DNMTs responsible for *ZNF154* promoter methylation, we performed siRNA-mediated knockdown of DNMT1, DNMT3A, and DNMT3B in OC cells. The knockdown efficiency was confirmed by qRT-PCR (Supplementary Fig. S1A, B). Interestingly, only knockdown of DNMT1 significantly decreased the methylation level of *ZNF154* and increased its expression in A2780 and SKOV3 cells (Fig. 3A, B). The more pronounced upregulation of ZNF154 in A2780 cells versus SKOV3 cells upon DNMT1 knockdown may be attributed primarily to its extremely low basal expression in A2780 cells relative to SKOV3 cells. Furthermore, treatment of OC cells with GSK3685032, a DNMT1-selective inhibitor, led to *ZNF154* promoter demethylation with concomitant transcriptional upregulation (Fig. 3C, D). Given the established role of UHRF1–DNMT1 interaction in sustaining DNA methylation patterns [33]. we also knocked down UHRF1 and observed a decrease in *ZNF154* PMR and an increase in ZNF154 expression (Fig. 3E, F and Supplementary Fig. S1C). Additionally, we employed a gain-of-function strategy in OC cells to explore the role of ZNF154 in ovarian tumorigenesis (Fig. 3G, H). Strikingly, ZNF154 overexpression significantly inhibited OC cell proliferation, migration and invasion (Fig. 3I–L). The findings clearly indicate that ZNF154 is epigenetically regulated by DNMT1/UHRF1-mediated methylation and functions as a tumor suppressor in OC.

### Targeted demethylation activates ZNF154 expression and inhibits proliferation, migration, and invasion of OC cells

**Fig. 4:**
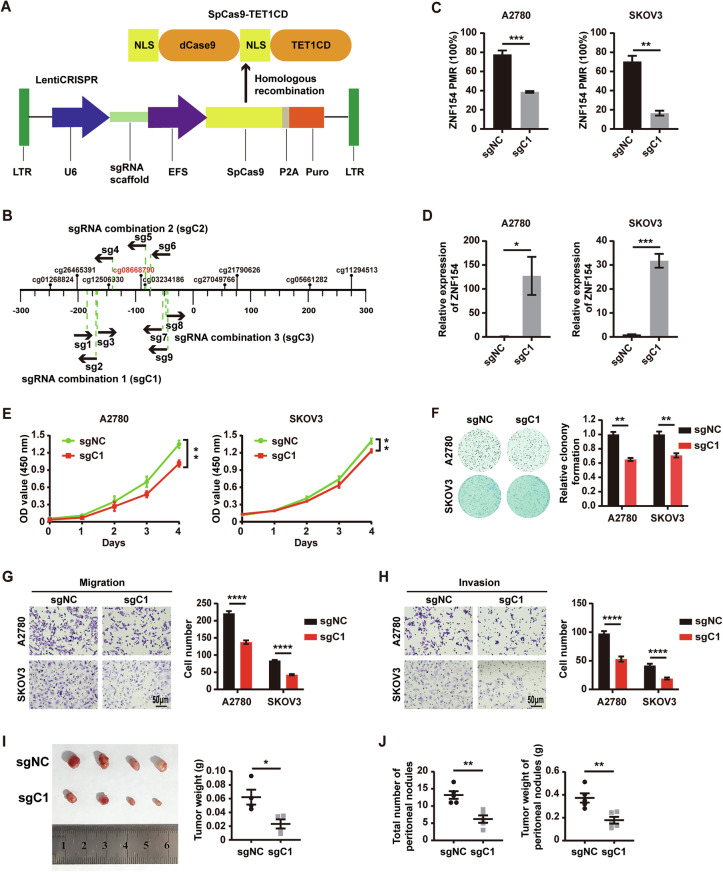
Targeted demethylation activates ZNF154 expression and inhibits proliferation, migration, and invasion of OC cells. **A** Schematic diagram illustrating the construction of the CRISPR/dCas9-TET1CD plasmid. **B** Diagram showing sgRNA target sites in *ZNF154* promoter. Arrowheads indicate the 3ʹ end of the sgRNA. **C** Bar plots showing *ZNF154* PMR in A2780 and SKOV3 cells expressing sgNC or sgC1 with the dCas9-TET1CD fusion protein. **D** Bar plots showing *ZNF154* mRNA levels in A2780 and SKOV3 cells expressing sgNC or sgC1 with the dCas9-TET1CD fusion protein. **E** CCK-8 assay assessing cell proliferation in cells expressing sgC1 or sgNC. **F** Colony formation assay in cells expressing sgC1 or sgNC (left panel), with accompanying quantitative analysis (right panel). **G** Transwell migration assay of cells expressing sgC1 or sgNC (left panel) and the accompanying quantitative analysis (right panel). **H** Transwell invasion assay of cells expressing sgC1 or sgNC (left panel) and the accompanying quantitative analysis (right panel). **I** Representative images and quantitative analysis of tumors isolated from mice injected with A2780 cells of the indicated genetic background. **J** Quantitative analysis of peritoneal tumor nodules derived from A2780 cells of the indicated genetic background. Data are presented as mean ± SEM. The Student’s *t* test, Welch’s *t* test, and two-way ANOVA were used for statistical comparisons as appropriate. *p < 0.05; **p < 0.01; ***p < 0.001; ****p < 0.0001; ns not significant. sgC1 combination of sgRNA1, sgRNA2, and sgRNA3, sgC2 combination of sgRNA4, sgRNA5, and sgRNA6, sgC3 combination of sgRNA7, sgRNA8, and sgRNA9, sgNC negative control sgRNA, LTR long terminal repeat, U6 U6 promoter, EFS EFS promoter, Puro puromycin resistance gene, SpCas9 *Streptococcus pyogenes* Cas9, NLS nuclear localization signal.

Given that ZNF154 was silenced by DNA methylation and functioned as a tumor suppressor in OC, we next sought to determine whether targeted demethylation could influence the tumor-suppressive function of ZNF154. To this end, we utilized a CRISPR/dCas9-TET1CD system to reactivate endogenous ZNF154 expression. This demethylation system is based on CRISPR/Cas9 platform. Briefly, the sequence encoding dCas9 was fused with TET1CD, replacing the Cas9 sequence in the CRISPR plasmid. Leveraging evidence that multiplexed sgRNA delivery can significantly enhance the reactivation of endogenous gene expression [34–36]. we designed 9 sgRNAs near the TSS and divided them into three groups (sgC1, sgC2 and sgC3) according to spatial distribution across the *ZNF154* CpG island (Fig. 4A, B). The activation efficiency of these groups was assessed by qRT-PCR. Remarkably, only sgC1 (the combination of sgRNAs 1–3) significantly increased *ZNF154* mRNA levels (Supplementary Fig. S2). Hence sgC1 was then selected for further study. As expected, OC cells infected with lentiviruses containing sgC1 exhibited a decrease in *ZNF154* PMR and an increase in ZNF154 expression (Fig. 4C, D). The CCK-8 and colony formation assays showed that ZNF154 demethylation inhibited the proliferation of OC cells (Fig. 4E, F). Moreover, demethylation of the *ZNF154* promoter impaired both migratory and invasive capabilities of OC cells (Fig. 4G, H).

To further confirm these results in vivo, we established both subcutaneous xenograft model and tumor metastasis model using BALB/c nude mice and NSG mice, respectively. Consistent with the in vitro results, tumors derived from A2780 wild-type cells in subcutaneous xenograft model were significantly larger than those derived from ZNF154 demethylation cells (Fig. 4I). Similarly, in the peritoneal metastasis model, mice intraperitoneally injected with A2780 wild-type cells showed significantly higher tumor burden than those receiving ZNF154 demethylation cells (Fig. 4J).

### *ROMO1* is a direct target of ZNF154

**Fig. 5:**
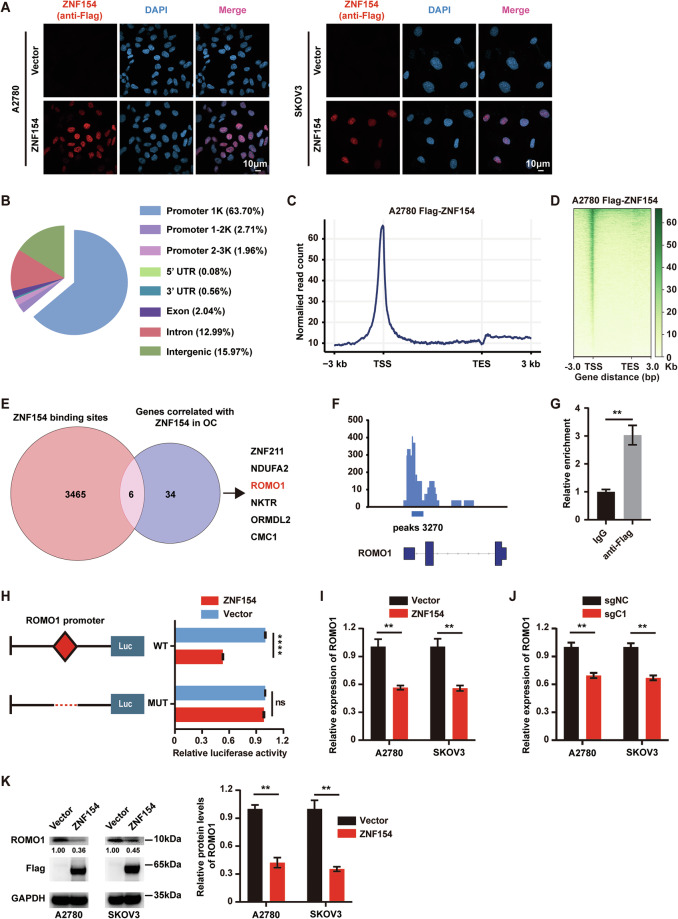
*ROMO1* is a direct target of ZNF154. **A** Immunofluorescence images showing the subcellular localization of ZNF154 protein. Scale bar, 10 μm. **B** Pie chart showing the genomic distribution of Flag-tagged CUT&Tag peaks in A2780 cells stably expressing Flag-ZNF154. **C** ZNF154 peak distribution (±3 kb from the TSS) at ZNF154-bound promoters. **D** ZNF154 CUT&Tag density heatmap (±3 kb from the TSS) in A2780 cells stably expressing Flag-ZNF154. Genes are sorted by density (high to low). **E** Venn diagram showing candidate target genes of ZNF154. **F** Diagram depicting ZNF154 binding to *ROMO1* promoter in A2780 cells stably expressing pLV-Flag-ZNF154. **G** ChIP-qPCR analysis of A2780 cells stably expressing Flag-ZNF154 using an anti-Flag antibody or control IgG antibody. **H** Luciferase reporter assay in A2780 cells stably overexpressing pLV-Vector or pLV-ZNF154 and transiently transfected with the ROMO1-Luc reporter plasmid (wild-type or mutant) for 48 h. **I**–**J** qRT-PCR analysis showing *ROMO1* expression in the indicated cells. **K** Representative immunoblots (left) and corresponding quantitative data (right) of the indicated proteins in the specified cell lines. Data are presented as mean ± SEM. Statistical significance was determined using a two-tailed Student’s *t*-test. ** *p* < 0.01; **** *p* < 0.0001; ns not significant. sgC1 combination of sgRNA1, sgRNA2, and sgRNA3, sgNC negative control sgRNA.

Like other ZFPs, ZNF154 is postulated to function as a nuclear transcription factor. Immunohistochemical analysis demonstrated predominant nuclear localization of ZNF154, a finding corroborated by immunofluorescence staining in OC cells with ectopic expression of Flag-ZNF154 (Figs. 2E and 5A). These observations substantiate its potential role as a transcriptional regulator. Thus, CUT&Tag analysis was used to identify direct downstream target genes of ZNF154. Given the unavailability of a validated ZNF154 antibody for ChIP, we performed Flag-based ChIP assays in A2780 cells stably expressing Flag-ZNF154. A total of 5204 ZNF154-specific binding peaks were identified, with most of the peaks (68.4%) localized to the promoter region of 3471 genes (Fig. 5B–D). To identify bona fide ZNF154 target genes, we intersected the initial 3471 candidate genes with those showing expression correlation with *ZNF154* in OC tissue (data from UALCAN database), narrowing down the candidates to 6 genes (Fig. 5E). Among these genes, we focused on *ROMO1* due to its known role in tumorigenesis [37–42]. Notably, IGV analysis revealed a ZNF154 binding peak at the *ROMO1* promoter (peak3270) (Fig. 5F). Further analysis of peak3270 showed that the most significant binding site between ZNF154 and the *ROMO1* promoter was the sequence CGGGCGGGGACGGAAGCGG. The binding of ZNF154 to *ROMO1* promoter was confirmed by ChIP-qPCR (Fig. 5G). To assess the functional impact of this binding, we performed luciferase reporter assay and found that ZNF154 repressed *ROMO1* promoter activity. This repression was attenuated by a mutation in the CGGGCGGGGACGGAAGCGG binding site (Fig. 5H). To further confirm the transcriptional repression of *ROMO1* by ZNF154, we assessed ROMO1 expression in both ZNF154-overexpressing cells and cells with ZNF154 demethylation. The results demonstrated that both ZNF154 overexpression and demethylation reduced ROMO1 expression (Fig. 5I–K).

### The effect of ZNF154 on OC growth and metastasis is mediated by ROMO1

**Fig. 6:**
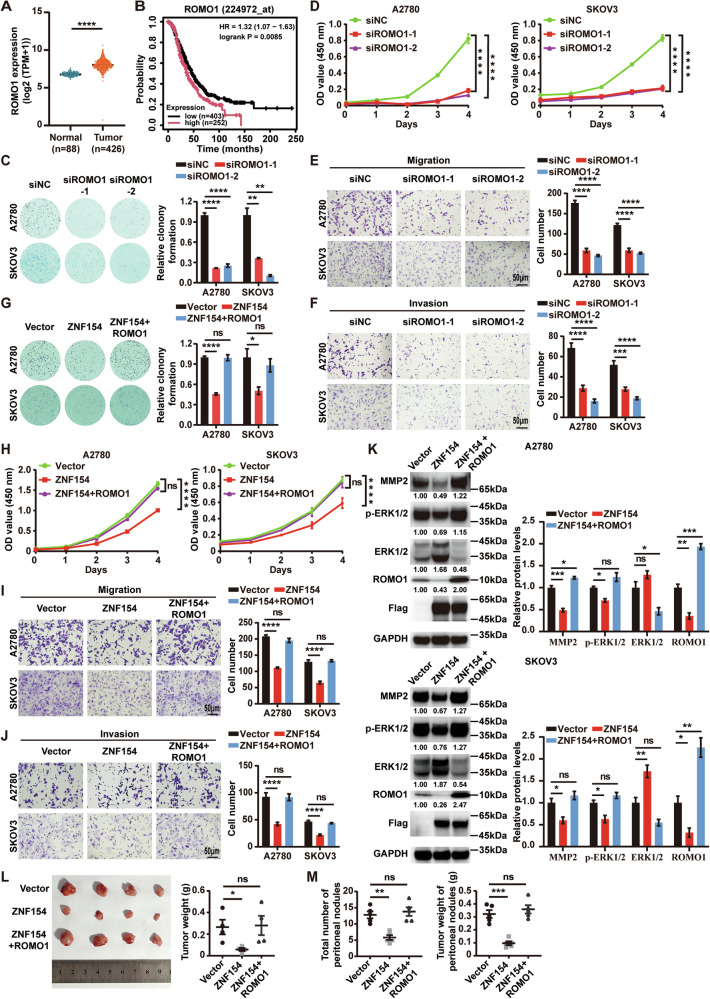
The effect of ZNF154 on OC growth and metastasis is mediated by ROMO1. **A** Analysis of *ROMO1* mRNA expression in OC tissues and normal tissues based on a combined dataset from TCGA and GTEx. **B** Kaplan–Meier survival analysis based on *ROMO1* mRNA expression in OC patients (*n* = 655) using the Kaplan–Meier Plotter tool. **C** Colony formation assay of A2780 and SKOV3 cells transfected with two distinct *ROMO1* siRNAs (left panel) with accompanying quantitative analysis (right panel). **D** CCK-8 assay assessing proliferation of A2780 and SKOV3 cells transfected with two distinct *ROMO1* siRNAs. **E** Transwell migration assay of A2780 and SKOV3 cells transfected with two distinct *ROMO1* siRNAs (left panel) with accompanying quantitative analysis (right panel). **F** Transwell invasion assay of A2780 and SKOV3 cells transfected with two distinct *ROMO1* siRNAs (left panel) with accompanying quantitative analysis (right panel). **G** Colony formation assay of the indicated stable cell lines (left panel) with accompanying quantitative analysis (right panel). **H** CCK-8 assay assessing proliferation of the indicated stable cell lines. **I** Transwell migration assay of the indicated stable cell lines (left panel) with accompanying quantitative analysis (right panel). **J** Transwell invasion assay of the indicated stable cell lines (left panel) with accompanying quantitative analysis (right panel). **K** Representative immunoblots (left) and corresponding quantitative data (right) of the indicated proteins in the specified cell lines. **L** Representative images and quantitative analysis of tumors isolated from mice injected with A2780 cells of the indicated genetic background. **M** Quantitative analysis of peritoneal tumor nodules derived from A2780 cells of the indicated genetic background. Data are presented as median (interquartile range) for non-normally distributed variables and mean ± SEM for normally distributed variables. Between-group comparisons were analyzed using the Mann–Whitney *U* test, one-way ANOVA and two-way ANOVA as appropriate. * *p* < 0.05; ** *p* < 0.01; *** *p* < 0.001; ns not significant.

Next, we focused on the role of ROMO1 in ovarian tumorigenesis. Bioinformatic analysis revealed elevated *ROMO1* mRNA abundance in OC specimens relative to non-malignant controls (Fig. 6A). Survival analysis revealed significantly worse overall survival in OC patients with elevated ROMO1 expression versus low-expressors (Fig. 6B). We then employed a loss-of-function strategy in A2780 and SKOV3 cells using siRNA. As expected, knockdown of ROMO1 attenuated proliferative, migratory, and invasive capacities of OC cells (Supplementary Fig. S3, Fig. 6C–F). To further investigate the functional interaction between ZNF154 and ROMO1, we overexpressed ROMO1 in ZNF154-overexpressing OC cells and carried out rescue experiments. The results revealed that ectopic expression of ROMO1 reversed the inhibition of proliferation, migration and invasion induced by upregulation of ZNF154 in OC cells (Fig. 6G–J). Previous studies have demonstrated that extracellular signal-regulated kinase (ERK) and matrix metalloproteinase 2 (MMP2) serve as critical downstream effectors of ROMO1, which facilitates the proliferation and metastasis of tumor cells by modulating intracellular levels of MMP2 and phosphorylated ERK [43, 44]. In this study, we found that ZNF154 overexpression not only impaired ROMO1 expression but also reduced the levels of MMP2 and phosphorylated ERK, while ectopic expression of ROMO1 rescued the expression of MMP2 and phosphorylated ERK (Fig. 6K). Consistent with these results, simultaneous overexpression of ROMO1 and ZNF154 reversed the tumor-suppressive effects of ZNF154 on tumor growth and metastasis in both subcutaneous xenograft and peritoneal metastasis models (Fig. 6L, M). These integrated findings establish ROMO1 as the primary downstream effector through which ZNF154 exerts tumor-suppressive effects in ovarian carcinogenesis.

### ZNF154 transcriptionally represses ROMO1 through KAP1

**Fig. 7:**
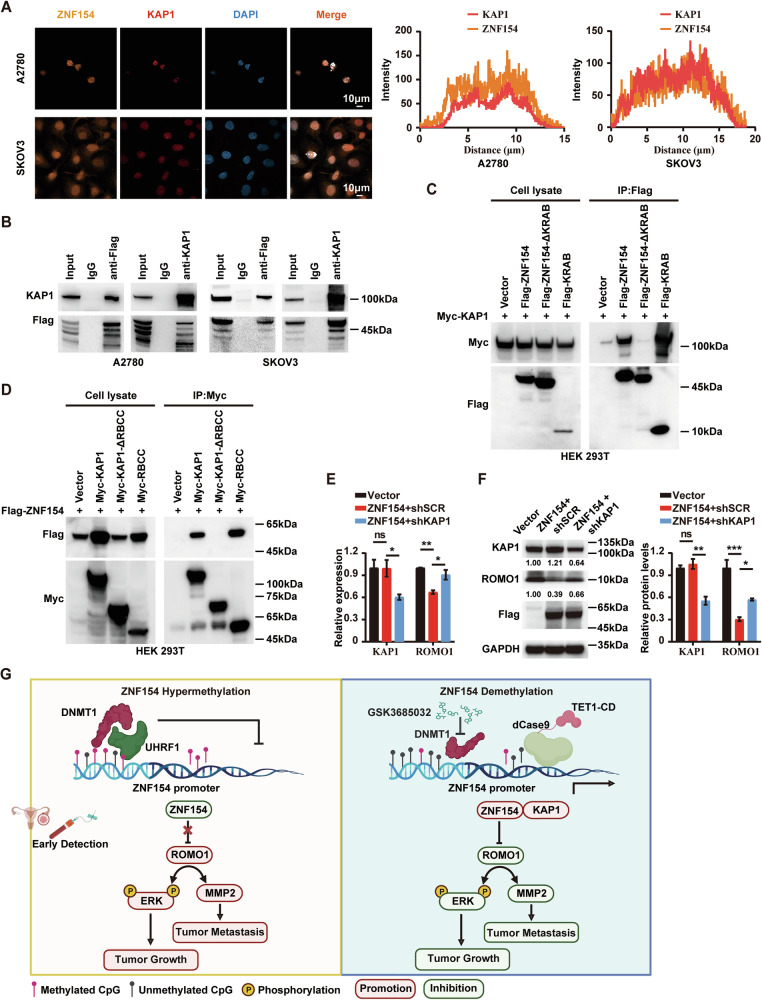
ZNF154 transcriptionally represses ROMO1 through KAP1. **A** ZNF154 and KAP1 colocalize in the nucleus. Immunofluorescence analysis was performed in A2780 and SKOV3 cells stably expressing Flag-ZNF154 using anti-ZNF154 and anti-KAP1 antibodies. Colocalization of ZNF154 and KAP1 was analyzed using ZEN v3.0 software. (Left panel: representative immunofluorescence images; Right panel: colocalization analysis). Scale bar, 10 μm. **B** ZNF154 interacts with KAP1. CoIP was performed in A2780 and SKOV3 cells stably expressing Flag-ZNF154 using anti-ZNF154 and anti-KAP1 antibodies. **C** KAP1 binds to the KRAB domain of ZNF154. CoIP was performed in HEK 293T cells cotransfected with Myc-KAP1 and Flag-ZNF154 truncation mutants. **D** ZNF154 binds to the RBCC domain of KAP1. CoIP was performed in HEK 293T cells cotransfected with Flag-ZNF154 and Myc-KAP1 truncation mutants. **E** Effect of KAP1 knockdown on *ROMO1* expression in A2780 cells stably expressing Flag-ZNF154, as assessed by qRT-PCR. **F** Representative immunoblots (left) and corresponding quantitative data (right) of the indicated proteins in the specified cell lines. **G** Schematic model depicting the role of ZNF154 in OC progression. Data are presented as mean ± SEM. Statistical significance was determined using one-way ANOVA. * *p* < 0.05; *** *p* < 0.001; ns not significant. ΔKRAB KRAB domain deletion mutant, ΔRBCC RBCC domain deletion mutant.

We aimed to decipher the molecular mechanism through which ZNF154 regulates ROMO1 expression. Consistent with its membership in the KRAB-ZFPs family, ZNF154 protein contains a KRAB domain at the N-terminus, which typically interacts with KAP1 as a scaffold to recruit chromatin-modifying factors such as heterochromatin protein 1, SET domain bifurcated 1 and histone deacetylases to mediate transcriptional repression [45–47]. However, the interaction between ZNF154 and KAP1 has not been well characterized. To investigate whether ZNF154 interacted with KAP1, we performed immunofluorescence analysis in OC cells and found that ZNF154 and KAP1 colocalized in the nucleus (Fig. 7A). Furthermore, coIP assays revealed that Flag-ZNF154 was present in KAP1 complexes and conversely, KAP1 was detected in Flag-ZNF154 complexes immunoprecipitated from A2780 and SKOV3 cells stably expressing Flag-ZNF154 (Fig. 7B). To further clarify the domains responsible for their interaction, we generated a series of truncation mutants of ZNF154 and KAP1 followed by immunofluorescence and coIP assays. Immunofluorescence analysis showed that both wild-type and all mutant forms of ZNF154, as well as both wild-type KAP1 and its ΔRBCC mutant, were predominantly localized to the nucleus (Supplementary Fig. S4A, B). Notably, the isolated RBCC domain localized to the cytoplasm, and its co-expression with ZNF154 induced a partial cytoplasmic shift of ZNF154 (Supplementary Fig. S4B, C). These findings are consistent with previous reports [48]. The coIP assays showed that ZNF154 bound with RBCC domain of KAP1, while the KRAB domain of ZNF154 was essential for its interaction with KAP1 (Fig. 7C, D). Next, to evaluate whether KAP1 was required for ZNF154-mediated downregulation of ROMO1, we assessed the expression of ROMO1 in ZNF154-overexpressing A2780 cells with KAP1 knockdown. Given the essential role of KAP1 in cell survival, we could only achieve its partial knockdown [11, 49–51]. Under this condition, the knockdown of KAP1 partially rescued ROMO1 expression, which had been repressed by ZNF154 (Fig. 7E, F). Collectively, these results demonstrate that ZNF154 interacts with KAP1 to form a transcriptional repression complex that negatively regulates ROMO1 expression.

## Discussion

Epigenetic dysregulation plays a pivotal role in cancer biology, particularly in aberrant gene expression profiles driven by aberrant DNA methylation, which is crucial for tumor initiation and progression [52]. In this study, we identified *ZNF154* as a DNA methylation-driven gene in OC, demonstrating that its hypermethylation leads to transcriptional silencing and correlates with poor prognosis. Our findings reveal that the DNMT1/UHRF1 complex is responsible for maintaining *ZNF154* hypermethylation. Furthermore, we demonstrated that ZNF154 functions as a tumor suppressor and targeted demethylation-mediated ZNF154 reactivation inhibits ovarian cancer cell malignancy. Mechanistically, we uncovered a novel molecular axis wherein ZNF154 recruits KAP1 to transcriptionally repress *ROMO1* (Fig. 7G). In vivo studies corroborated the tumor-suppressive effects of ZNF154 reactivation, underscoring its therapeutic potential. These findings may contribute to the evaluation of *ZNF154* hypermethylation as a prognostic biomarker and prompt the investigation of epigenetic reprogramming in OC therapy. Targeting DNA methylation to restore ZNF154 expression may represent a potential strategy to improve treatment outcomes.

Dysregulated DNA methylome is a hallmark of cancer and represents a promising therapeutic target in cancer therapy [53]. Currently, the most widely used drugs targeting aberrant DNA methylation are DNMTs inhibitors (DNMTis), which inhibit DNMTs and reverse hypermethylation [53]. In this study, we identified that ZNF154 acts as a tumor suppressor whose expression is epigenetically silenced via hypermethylation mediated by the DNMT1/UHRF1 complex. This mechanistic insight suggests that the *ZNF154* promoter might be particularly susceptible to DNMT1-selective inhibition. However, translating this molecular vulnerability into effective therapy faces major clinical challenges. The efficacy of DNMTis in solid tumors is constrained by dose-limiting toxicity and suboptimal drug delivery [54–57]. Notably, a recent trial of low-dose DNMTis plus pembrolizumab demonstrated good tolerability but limited clinical benefit in OC [58, 59]. Meanwhile, by employing a CRISPR/dCas9-based demethylation approach that efficiently demethylated target gene promoter [7, 28, 60]. we successfully reactivated ZNF154 expression. This reactivation significantly inhibited OC cell migration and invasion in vitro and in vivo. These findings provide a theoretical foundation for considering ZNF154 as a candidate of interest in the development of more precise epigenetic therapeutic strategies for OC, warranting further investigation into its functional role and therapeutic potential.

Given the early evidence indicating a potential role for *ZNF154* in OC diagnosis [23–27]. we quantified its promoter methylation in serum cfDNA from patients and controls using a cost-effective MethyLight assay [61, 62]. This analysis revealed that *ZNF154* promoter methylation in cfDNA demonstrates a sensitivity of 81.25% and a specificity of 89.66% for OC diagnosis, with comparable performance in early-stage detection (sensitivity 87.50%, specificity 82.76%). Owing to the retrospective design and limited sample size of this study, these findings will require validation in larger prospective cohorts. Notably, the presence of *ZNF154* promoter hypermethylation in other epithelial tumors limits its specificity for OC [21–24, 63]. Given that integrating multiple biomarkers has been shown to improve diagnostic performance for OC, *ZNF154* methylation may be more suitable as a component of an integrated biomarker panel [27, 64].

As a member of the KRAB-ZFPs family, ZNF154 contains an N-terminal KRAB domain that mediates protein-protein interactions and C-terminal zinc finger repeats responsible for sequence-specific DNA binding [65–68]. Although KRAB-ZFPs primarily exert their functions through zinc finger domain-dependent targeting of gene promoters [65]. ZNF154 has been largely overlooked in cancer research. Previous studies have predominantly focused on its hypermethylation in tumor tissues, with limited exploration of its functional roles [22, 23, 25, 27, 28]. Notably, the contribution of ZNF154 to OC pathogenesis remains virtually unexplored. We demonstrated the tumor suppressor properties of ZNF154 in OC via in vitro and in vivo experiments. To explore its mechanism, we performed CUT&Tag analysis, identifying 3471 potential target genes. Intersecting these with gene expression profiles revealed that *ROMO1* acts as a direct target of ZNF154. Further validation by qRT-PCR and Western blotting revealed that ectopic ZNF154 expression significantly inhibited *ROMO1* transcription and translation.

ROMO1 is a key mitochondrial modulator regulating cellular reactive oxygen species (ROS) generation and mitochondrial homeostasis [37]. Accumulating evidence has revealed the important role of ROMO1 in tumor progression. ROMO1-derived ROS are essential for cancer cell proliferation, since ROMO1 downregulation inhibits cell growth and induces apoptosis [38–40, 43]. Conversely, ROMO1 upregulation promotes the proliferation and metastasis of cancer cells [41, 42]. Aberrant ROMO1 levels correlate with poor clinical outcomes in multiple malignancies, underscoring its tumorigenic functions [69]. Notably, a recent study revealed that ROMO1 overexpression protects mitochondrial proteins from oxidative damage during aging, thereby enhancing mitochondrial function and delaying functional decline [70]. The pro-tumorigenic role of ROMO1 in cancer versus its protective role in aging suggests that it functions as a context-dependent redox regulator. This functional dichotomy implies that its role may vary across different cancer types.

Although a recent study indicated that ROMO1 overexpression enhances OC cell proliferation, its functional roles in OC pathogenesis and metastasis remain incompletely defined [69]. In this study, we revealed the oncogenic properties of ROMO1 in promoting the proliferation, migration and invasion of OC cells. Previous studies have shown that ROMO1-driven elevation of ROS can activate the ERK1/2 pathway [43]. This activation not only increases phosphorylated ERK levels but also upregulates MMP2 expression by enhancing the transcriptional activity of Sp1 [43, 71]. Additionally, ROS could also upregulate MMP2 by increasing the transcriptional activity of FoxO3a [72]. ERK is a key mitogen-activated protein kinase (MAPK) in the RAS/RAF/MAPK signaling pathway that mediates cell proliferation and migration when phosphorylated [73, 74]. While MMP2 is a matrix metalloproteinase that facilitates extracellular matrix degradation to promote invasion [75, 76]. Therefore, the tumor-suppressive function of ZNF154 is achieved by suppressing ROMO1 expression, which leads to reduced levels of phosphorylated ERK and MMP2. Furthermore, we demonstrated the interaction between the KRAB domain of ZNF154 and RBCC domain of KAP1 by coIP assay. This ZNF154–KAP1 interaction was crucial for forming a silencing complex that mediates the transcriptional repression of ROMO1, as KAP1 knockdown rescued ROMO1 expression. Taken together, these findings establish the ZNF154/KAP1–ROMO1–ERK/MMP2 axis as a critical pathway for ZNF154-mediated inhibition of tumor growth and metastasis, providing a mechanistic basis for future therapeutic exploration.

In summary, this study identifies *ZNF154* as a tumor suppressor gene in OC whose expression is silenced by DNA methylation. We demonstrated that ZNF154 was hypermethylated and downregulated in OC, with DNMT1 and UHRF1 mediating its silencing. Using CRISPR-based epigenetic editing, we reactivated ZNF154, which inhibited OC cell proliferation and invasion via the ROMO1–ERK/MMP2 pathway. Furthermore, *ZNF154* promoter hypermethylation in OC tissues is associated with poor clinical outcomes. Additionally, *ZNF154* promoter methylation in cfDNA is a candidate for the detection of OC, with its potential utility alongside other markers awaiting validation in larger cohorts. These findings implicate aberrant *ZNF154* methylation in OC pathogenesis, suggesting it merits further investigation both as a prognostic marker and a therapeutic target.

## Supplementary information

Supplementary Figures and Tables

Original Western Blot

## Data Availability

Data used in the preparation of this manuscript are available within the article and supplementary information file. There are no restrictions on data access. Additional experimental details and material requests may be directed to the corresponding author for legitimate scientific purposes.
