# Supplementary material for: Hypermethylation of ZNF154 promotes malignant potential of ovarian cancer cells by diminishing ZNF154/KAP1-mediated ROMO1 repression
Source: Cell Death Dis. 2026 May 2;17(1):582. doi: 10.1038/s41419-026-08823-w (PMC13280250; doi:10.1038/s41419-026-08823-w)
Supplement: Supplementary file 1 — Supplementary Figures and Tables [file 41419_2026_8823_MOESM1_ESM.docx]

**Supplementary Information for**

**Hypermethylation of ZNF154 promotes malignant potential of ovarian cancer cells by diminishing ZNF154/KAP1-mediated ROMO1 repression**

**Running title: ZNF154 inhibits ovarian cancer via repressing ROMO1**

Mingbiao Wei^#^, Yuxia Xu^#^, Ling Deng^#^, Wei Wei, Dongyi Ling, Shanshan Zhen, Ran Zhou, Wenjian Cen, Xu Zhang, Mayan Huang, Jundong Li^*^, Shumei Yan^*^, Qin Li^*^, Ziming Du^*^

^#^These authors contributed equally to this work

^*^Correspondence: duzm8@sysu.edu.cn; liqin63@mail.sysu.edu.cn; yanshm@sysucc.org.cn; lijd@sysucc.org.cn

**Table of Contents**

[Figure S1. Knockdown of DNMTs and UHRF1 in OC cells. 2](#_Toc201650759)

[Figure S2. The CRISPR/dCas9-TET1CD system reactivates ZNF154 expression. 3](#_Toc201650760)

[Figure S3. Knockdown of ROMO1 in OC cells. 4](#_Toc201650760)

Figure S4. Subcellular localization of KAP1 and ZNF154 mutants[. 5](#_Toc201650760)

[Table S1. Clinical raw data of cohort I 6](#_Toc201650761)

[Table S2. Clinical raw data of cohort II 10](#_Toc201650762)

[Table S3. Primers and probes used in MethyLight assay 12](#_Toc201650763)

[Table S4. The sequences of sgRNAs and shRNAs 13](#_Toc201650764)

[Table S5. Primers for qRT-PCR 14](#_Toc201650765)


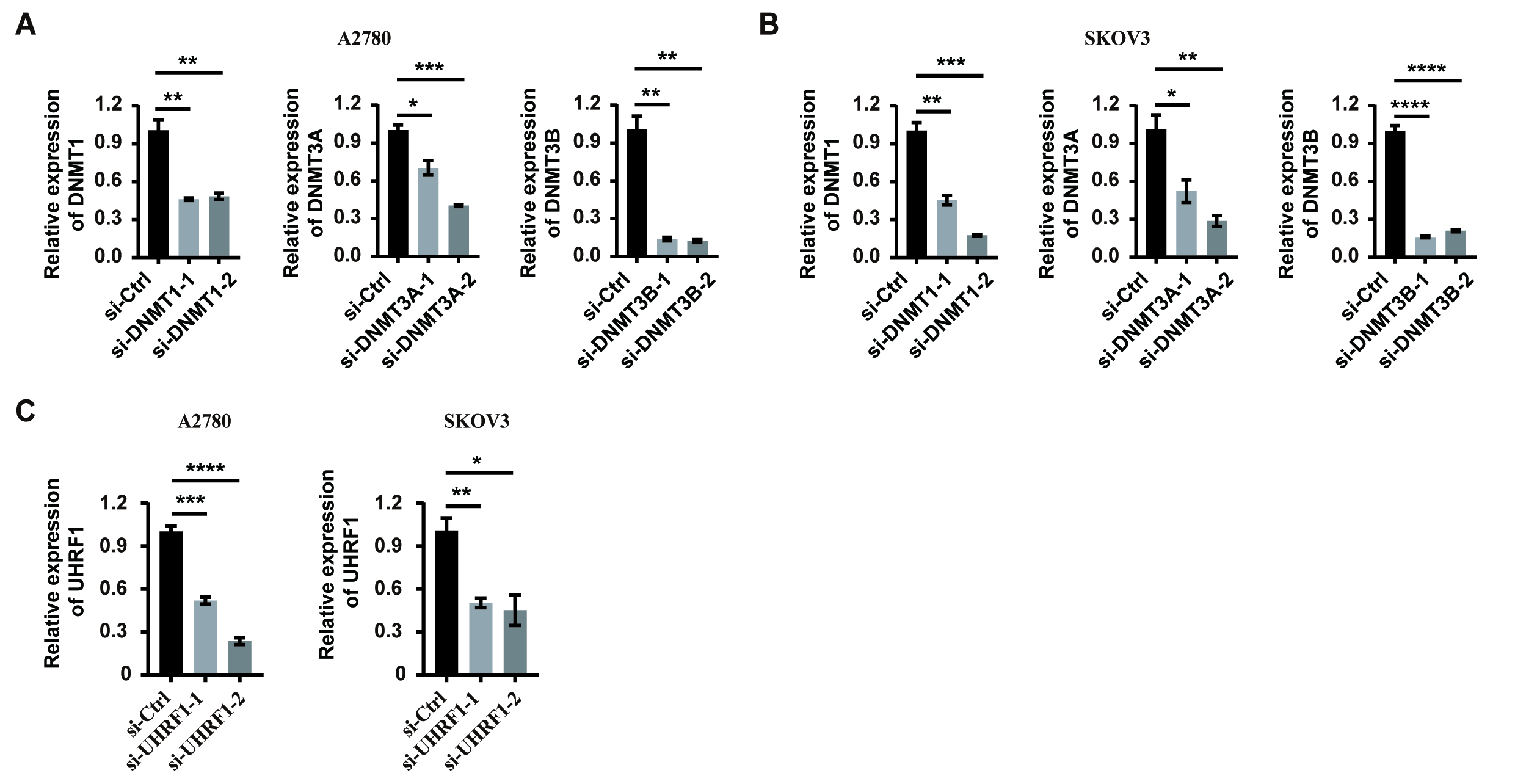


Figure S1. Knockdown of DNMTs and UHRF1 in OC cells. (A and B) qRT-PCR analysis of DNMT1, DNMT3A, and DNMT3B expression in A2780 and SKOV3 cells post-knockdown. (C) qRT-PCR analysis of UHRF1 expression in A2780 and SKOV3 cells following UHRF1 knockdown. Data are presented as mean ± SEM. Statistical significance was determined using one-way ANOVA. * *p* < 0.05; ** *p* < 0.01; *** *p* < 0.001; **** *p* < 0.0001.


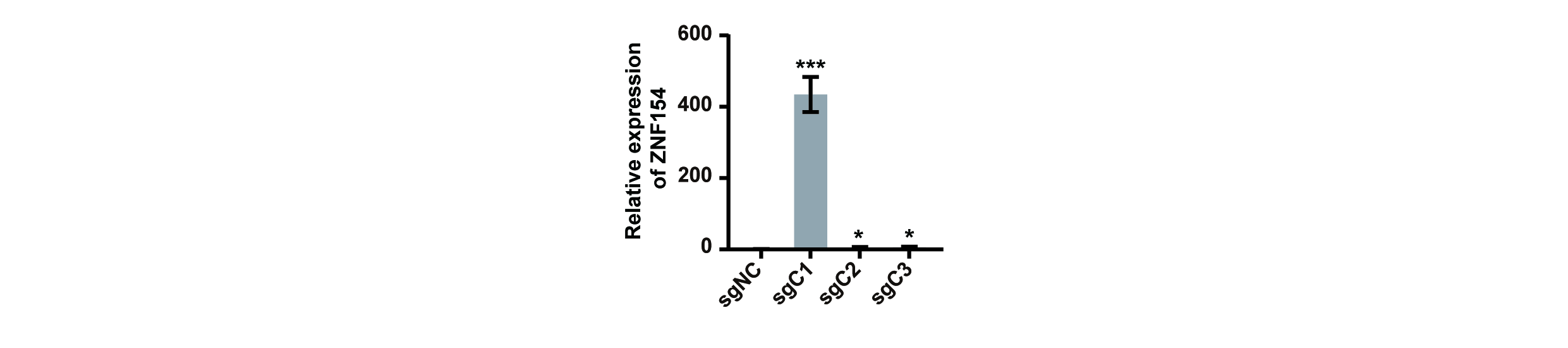


Figure S2. The CRISPR/dCas9-TET1CD system reactivates ZNF154 expression. qRT-PCR analysis of ZNF154 expression in A2780 cells stably expressing the indicated sgRNA and dCas9-TET1CD fusion protein. Data are presented as mean ± SEM. Statistical significance was determined using one-way ANOVA. * *p* < 0.05; *** *p* < 0.001; sgC1, the combination of sgRNA1, sgRNA2 and sgRNA3; sgC2, the combination of sgRNA4, sgRNA5 and sgRNA6; sgC3, the combination of sgRNA7, sgRNA8 and sgRNA9.


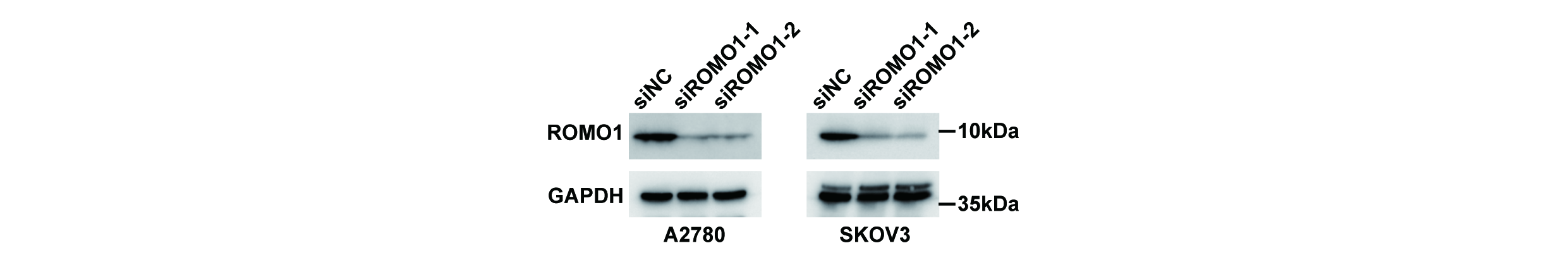


Figure S3. Knockdown of ROMO1 in OC cells. Western blot analysis showing expression of the indicated proteins in A2780 and SKOV3 cells transfected with *ROMO1* siRNAs.


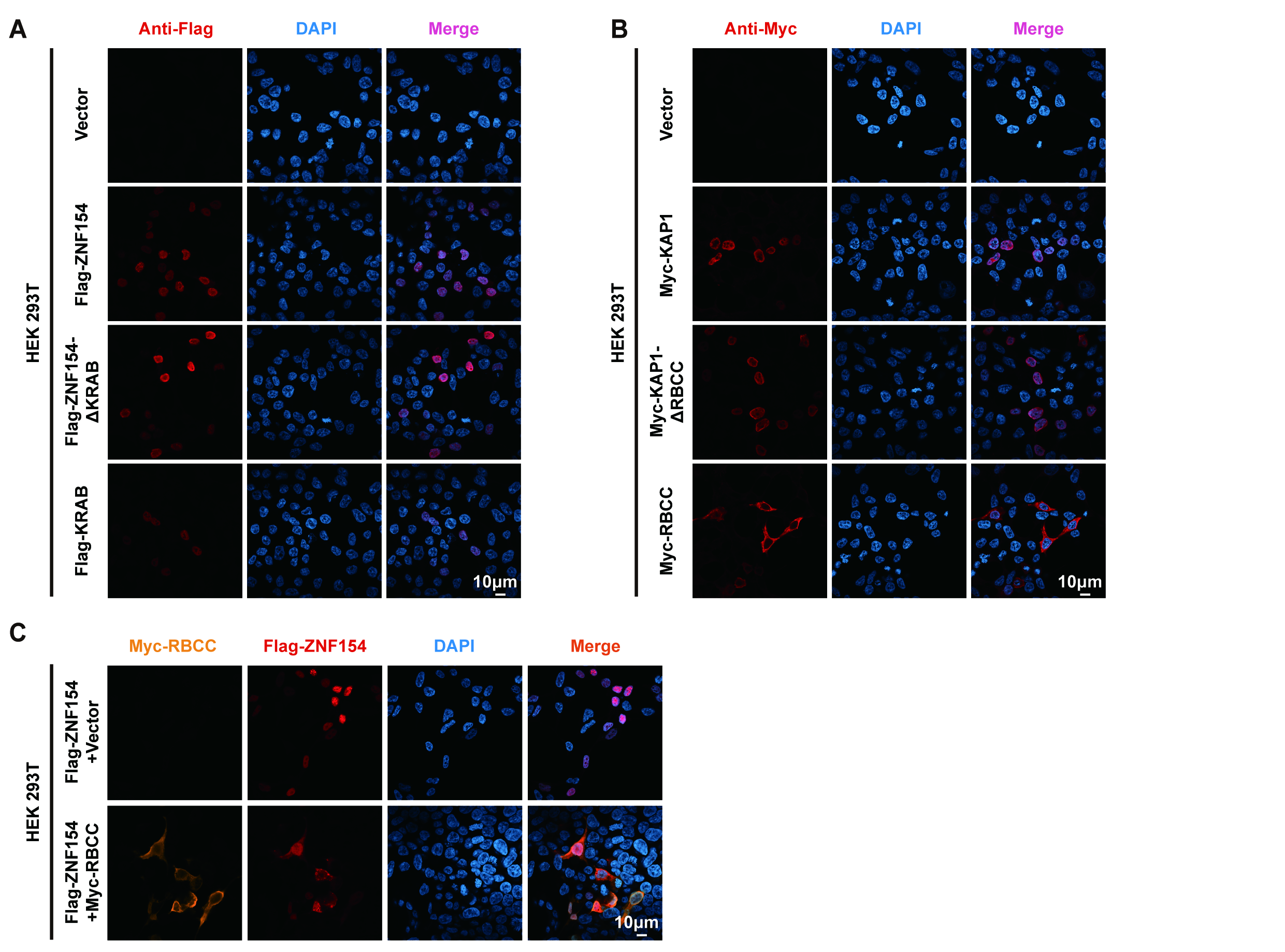


Figure S4. Subcellular localization of KAP1 and ZNF154 mutants. (A) Immunofluorescence analysis was performed in 293T cells transfected with ZNF154 mutants using an anti-Flag antibody. (B) Immunofluorescence analysis was performed in 293T cells transfected with KAP1 mutants using an anti-Myc antibody. (C) Immunofluorescence analysis was performed in 293T cells transfected with RBCC domain and ZNF154 using anti-Flag and anti-Myc antibodies. Scale bar, 10 μm.

Table S1. Clinical raw data of cohort I

| Sample ID | Gender | Age | TNM stage | Survival (0=survival, 1=death) | Survial time (months) | PMR level | IHC score |
| --- | --- | --- | --- | --- | --- | --- | --- |
| T1 | Female | 47 | Ⅰ | 0 | 41.3 | 47.35 | 6 |
| T2 | Female | 40 | Ⅰ | 0 | 3.9 | 51.69 | 0 |
| T3 | Female | 26 | Ⅰ | 0 | 35.6 | 35.19 | 1 |
| T4 | Female | 46 | Ⅰ | 0 | 21.5 | 75.55 | 2 |
| T5 | Female | 49 | Ⅰ | 0 | 0.2 | 33.93 | 0 |
| T6 | Female | 47 | Ⅱ | 0 | 25.5 | 51.35 | 2 |
| T7 | Female | 54 | Ⅳ | 1 | 5.5 | 36.45 | 0 |
| T8 | Female | 60 | Ⅲ | 1 | 19.0 | 28.36 | 2 |
| T9 | Female | 71 | Ⅳ | 0 | 68.1 | 17.66 | 4 |
| T10 | Female | 55 | Ⅲ | 0 | 54.7 | 31.67 | 0 |
| T11 | Female | 66 | Ⅰ | 0 | 42.1 | 7.75 | 6 |
| T12 | Female | 60 | Ⅰ | 0 | 40.1 | 28.14 | 2 |
| T13 | Female | 55 | Ⅲ | 1 | 39.4 | 30.85 | 0 |
| T14 | Female | 57 | Ⅱ | 0 | 42.2 | 52.58 | 0 |
| T15 | Female | 29 | Ⅰ | 0 | 77.1 | 32.25 | 2 |
| T16 | Female | 66 | Ⅰ | 0 | 38.7 | 12.91 | 2 |
| T17 | Female | 59 | Ⅰ | 0 | 15.2 | 53.26 | 1 |
| T18 | Female | 56 | Ⅱ | 0 | 13.0 | 37.71 | 0 |
| T19 | Female | 45 | Ⅰ | 0 | 38.8 | 47.85 | 1 |
| T20 | Female | 44 | Ⅰ | 0 | 42.3 | 39.53 | 2 |
| T21 | Female | 69 | Ⅱ | 0 | 38.5 | 15.44 | 6 |
| T22 | Female | 49 | Ⅱ | 0 | 38.2 | 70.77 | 0 |
| T23 | Female | 47 | Ⅱ | 0 | 47.6 | 26.52 | 0 |
| T24 | Female | 45 | Ⅰ | 0 | 46.8 | 12.78 | 1 |
| T25 | Female | 33 | Ⅰ | 0 | 2.8 | 13.49 | 4 |
| T26 | Female | 55 | Ⅱ | 0 | 22.6 | 51.46 | 2 |
| T27 | Female | 53 | Ⅰ | 0 | 73.7 | 14.98 | 1 |
| T28 | Female | 59 | Ⅲ | 0 | 68.9 | 2.86 | 0 |
| T29 | Female | 52 | Ⅳ | 0 | 60.5 | 43.99 | 4 |
| T30 | Female | 76 | Ⅰ | 1 | 2.2 | 39.61 | 2 |
| T31 | Female | 64 | Ⅳ | 0 | 93.7 | 6.87 | 4 |
| T32 | Female | 61 | Ⅳ | 1 | 55.0 | 17.83 | 2 |
| T33 | Female | 48 | Ⅱ | 0 | 93.4 | 11.12 | 2 |
| T34 | Female | 44 | Ⅲ | 1 | 37.2 | 29.04 | 0 |
| T35 | Female | 55 | Ⅲ | 1 | 89.3 | 72.44 | 1 |
| T36 | Female | 55 | Ⅲ | 0 | 82.5 | 51.20 | 0 |
| T37 | Female | 43 | Ⅲ | 1 | 13.6 | 50.58 | 2 |
| T38 | Female | 61 | Ⅲ | 1 | 43.0 | 70.83 | 4 |
| T39 | Female | 49 | Ⅲ | 1 | 15.4 | 60.81 | 2 |
| T40 | Female | 55 | Ⅳ | 1 | 25.5 | 50.66 | 1 |
| T41 | Female | 37 | Ⅳ | 0 | 4.4 | 32.80 | 2 |
| T42 | Female | 57 | Ⅳ | 1 | 7.5 | 64.49 | 0 |
| T43 | Female | 54 | Ⅳ | 0 | 62.9 | 2.59 | 3 |
| T44 | Female | 48 | Ⅲ | 1 | 48.7 | 29.41 | 1 |
| T45 | Female | 69 | Ⅲ | 1 | 57.1 | 33.74 | 1 |
| T46 | Female | 44 | Ⅲ | 1 | 28.3 | 57.10 | 2 |
| T47 | Female | 38 | Ⅲ | 0 | 65.8 | 44.46 | 3 |
| T48 | Female | 69 | Ⅲ | 0 | 5.8 | 38.80 | 0 |
| T49 | Female | 60 | Ⅲ | 0 | 53.6 | 51.55 | 3 |
| T50 | Female | 61 | Ⅱ | 0 | 51.4 | 17.61 | 2 |
| T51 | Female | 54 | Ⅳ | 1 | 51.7 | 43.30 | 0 |
| T52 | Female | 69 | Ⅲ | 0 | 53.5 | 24.31 | 2 |
| T53 | Female | 39 | Ⅲ | 1 | 32.8 | 50.19 | 1 |
| T54 | Female | 43 | Ⅳ | 1 | 62.6 | 78.43 | 0 |
| T55 | Female | 54 | Ⅳ | 0 | 35.3 | 31.78 | 1 |
| T56 | Female | 61 | Ⅲ | 0 | 62.6 | 44.84 | 0 |
| T57 | Female | 49 | Ⅳ | 0 | 37.6 | 50.76 | 3 |
| T58 | Female | 45 | Ⅲ | 1 | 39.5 | 45.39 | 1 |
| T59 | Female | 40 | Ⅲ | 0 | 67.6 | 42.57 | 4 |
| T60 | Female | 39 | Ⅳ | 1 | 13.1 | 21.46 | 0 |
| T61 | Female | 50 | Ⅲ | 1 | 46.9 | 44.49 | 1 |
| T62 | Female | 42 | Ⅳ | 1 | 22.3 | 31.31 | 0 |
| T63 | Female | 55 | Ⅲ | 0 | 66.9 | 63.95 | 1 |
| T64 | Female | 52 | Ⅱ | 1 | 58.9 | 24.07 | 2 |
| T65 | Female | 47 | Ⅳ | 1 | 23.9 | 79.55 | 2 |
| T66 | Female | 52 | Ⅳ | 0 | 64.4 | 20.70 | 0 |
| T67 | Female | 60 | Ⅲ | 0 | 50.0 | 36.72 | 3 |
| T68 | Female | 47 | Ⅳ | 1 | 40.7 | 41.86 | 4 |
| T69 | Female | 71 | Ⅱ | 0 | 13.6 | 24.81 | 0 |
| T70 | Female | 71 | Ⅲ | 1 | 13.3 | 2.14 | 1 |
| T71 | Female | 45 | Ⅰ | 0 | 47.7 | 2.60 | 2 |
| T72 | Female | 65 | Ⅳ | 0 | 54.7 | 38.23 | 1 |
| T73 | Female | 49 | Ⅳ | 1 | 23.4 | 20.79 | 1 |
| T74 | Female | 61 | Ⅲ | 1 | 15.9 | 50.31 | 1 |
| T75 | Female | 78 | Ⅱ | 0 | 41.3 | 24.64 | 0 |
| T76 | Female | 49 | Ⅳ | 1 | 26.5 | 30.41 | 1 |
| T77 | Female | 49 | Ⅲ | 0 | 30.0 | 21.91 | 1 |
| T78 | Female | 52 | Ⅳ | 0 | 39.6 | 74.80 | 0 |
| T79 | Female | 62 | Ⅲ | 1 | 15.4 | 18.77 | 1 |
| T80 | Female | 61 | Ⅲ | 0 | 60.4 | 4.10 | 1 |
| T81 | Female | 65 | Ⅳ | 1 | 22.5 | 4.85 | 6 |
| T82 | Female | 54 | Ⅳ | 1 | 59.7 | 14.93 | 1 |
| T83 | Female | 52 | Ⅲ | 1 | 27.9 | 17.69 | 6 |
| T84 | Female | 48 | Ⅰ | 0 | 49.2 | 2.35 | 2 |
| T85 | Female | 55 | Ⅳ | 0 | 60.2 | 39.44 | 1 |
| T86 | Female | 42 | Ⅳ | 0 | 28.1 | 21.92 | 4 |
| T87 | Female | 56 | Ⅲ | 1 | 25.0 | 8.40 | 0 |
| T88 | Female | 60 | Ⅲ | 0 | 42.1 | 13.47 | 1 |
| T89 | Female | 61 | Ⅲ | 1 | 52.9 | 16.32 | 2 |
| T90 | Female | 35 | Ⅱ | 0 | 60.7 | 1.18 | 4 |
| T91 | Female | 58 | Ⅱ | 0 | 4.6 | 35.98 | 2 |
| T92 | Female | 44 | Ⅰ | 0 | 49.5 | 25.50 | 2 |
| T93 | Female | 61 | Ⅱ | 1 | 31.2 | 32.52 | 3 |
| T94 | Female | 39 | Ⅰ | 0 | 41.7 | 7.76 | 4 |
| T95 | Female | 43 | Ⅱ | 0 | 53.7 | 11.22 | 1 |
| T96 | Female | 44 | Ⅱ | 0 | 51.6 | 19.79 | 1 |
| T97 | Female | 55 | Ⅱ | 0 | 14.6 | 9.94 | 2 |
| T98 | Female | 56 | Ⅱ | 0 | 20.3 | 12.25 | 1 |
| T99 | Female | 60 | Ⅱ | 0 | 33.1 | 42.87 | 1 |
| T100 | Female | 17 | Ⅱ | 0 | 8.6 | 25.54 | 0 |
| T101 | Female | 52 | Ⅰ | 0 | 31.7 | 29.40 | 0 |
| T102 | Female | 47 | Ⅰ | 0 | 12.9 | 53.00 | 1 |
| T103 | Female | 50 | Ⅱ | 0 | 23.8 | 47.98 | 2 |
| T104 | Female | 43 | Ⅳ | 1 | 45.8 | 17.46 | 1 |
| T105 | Female | 46 | Ⅲ | 0 | 66.0 | 11.46 | 1 |
| T106 | Female | 59 | Ⅳ | 0 | 60.0 | 21.91 | 6 |
| T107 | Female | 47 | Ⅳ | 0 | 45.9 | 11.18 | 1 |
| T108 | Female | 72 | Ⅲ | 0 | 44.8 | 3.59 | 6 |
| T109 | Female | 47 | Ⅱ | 0 | 25.0 | 28.57 | 0 |
| T110 | Female | 49 | Ⅲ | 0 | 42.7 | 34.33 | 1 |
| T111 | Female | 54 | Ⅲ | 1 | 19.4 | 28.45 | 2 |
| T112 | Female | 73 | Ⅲ | 0 | 50.0 | 35.67 | 0 |
| T113 | Female | 51 | Ⅳ | 1 | 7.7 | 28.89 | 1 |
| T114 | Female | 62 | Ⅳ | 1 | 52.1 | 22.42 | 2 |
| T115 | Female | 59 | Ⅳ | 0 | 44.3 | 8.34 | 3 |
| T116 | Female | 62 | Ⅳ | 1 | 45.7 | 33.94 | 2 |
| T117 | Female | 47 | Ⅲ | 1 | 33.8 | 2.83 | 0 |
| T118 | Female | 52 | Ⅲ | 0 | 48.1 | 22.34 | 0 |
| T119 | Female | 54 | Ⅲ | 0 | 57.3 | 52.42 | 0 |
| T120 | Female | 60 | Ⅲ | 1 | 8.0 | 33.74 | 0 |
| T121 | Female | 47 | Ⅳ | 0 | 63.3 | 7.87 | 3 |
| T122 | Female | 40 | Ⅳ | 0 | 53.1 | 44.49 | 2 |
| N1 | Female | 47 |  |  |  | 4.11 |  |
| N2 | Female | 40 |  |  |  | 2.77 | 9 |
| N3 | Female | 26 |  |  |  | 16.16 |  |
| N4 | Female | 46 |  |  |  | 11.64 |  |
| N5 | Female | 49 |  |  |  | 0.89 | 12 |
| N6 | Female | 47 |  |  |  | 3.38 | 12 |
| N7 | Female | 54 |  |  |  | 18.46 |  |
| N8 | Female | 60 |  |  |  | 9.28 |  |
| N9 | Female | 71 |  |  |  | 1.15 |  |
| N10 | Female | 55 |  |  |  | 3.93 |  |
| N11 | Female | 66 |  |  |  | 6.44 |  |
| N12 | Female | 60 |  |  |  | 3.77 |  |
| N13 | Female | 55 |  |  |  | 1.22 |  |
| N14 | Female | 57 |  |  |  | 3.77 |  |
| N15 | Female | 29 |  |  |  | 2.84 | 4 |
| N16 | Female | 66 |  |  |  | 3.79 |  |
| N17 | Female | 59 |  |  |  | 1.57 | 4 |
| N18 | Female | 56 |  |  |  | 3.86 | 6 |
| N19 | Female | 45 |  |  |  | 3.60 | 9 |
| N20 | Female | 44 |  |  |  | 3.32 |  |
| N21 | Female | 69 |  |  |  | 0.90 |  |
| N22 | Female | 49 |  |  |  | 4.30 |  |
| N23 | Female | 47 |  |  |  | 1.08 | 12 |
| N24 | Female | 45 |  |  |  | 2.71 | 8 |
| N25 | Female | 33 |  |  |  | 6.10 |  |
| N26 | Female | 55 |  |  |  | 8.31 |  |
| N27 | Female | 53 |  |  |  | 2.36 | 12 |
| N28 | Female | 59 |  |  |  | 29.17 |  |
| N29 | Female | 52 |  |  |  | 1.51 |  |
| N30 | Female | 76 |  |  |  | 1.83 |  |

Abbreviations: PMR, percent of methylated reference.

Table S2. Clinical raw data of cohort II

| Sample ID | Gender | Age | TNM stage | PMR level |
| --- | --- | --- | --- | --- |
| S-T1 | Female | 56 | Ⅰ | 1.05 |
| S-T2 | Female | 26 | Ⅳ | 2.21 |
| S-T3 | Female | 47 | Ⅲ | 2.74 |
| S-T4 | Female | 74 | Ⅳ | 2.79 |
| S-T5 | Female | 59 | Ⅲ | 3.52 |
| S-T6 | Female | 79 | Ⅳ | 3.75 |
| S-T7 | Female | 26 | Ⅲ | 4.48 |
| S-T8 | Female | 21 | Ⅰ | 4.62 |
| S-T9 | Female | 63 | Ⅱ | 4.99 |
| S-T10 | Female | 58 | Ⅲ | 5.11 |
| S-T11 | Female | 44 | Ⅳ | 5.15 |
| S-T12 | Female | 58 | Ⅳ | 5.99 |
| S-T13 | Female | 43 | Ⅳ | 6.00 |
| S-T14 | Female | 58 | Ⅲ | 6.17 |
| S-T15 | Female | 67 | Ⅲ | 6.21 |
| S-T16 | Female | 60 | Ⅲ | 6.30 |
| S-T17 | Female | 55 | Ⅲ | 7.20 |
| S-T18 | Female | 54 | Ⅲ | 7.93 |
| S-T19 | Female | 48 | Ⅲ | 8.06 |
| S-T20 | Female | 55 | Ⅲ | 8.79 |
| S-T21 | Female | 48 | Ⅳ | 9.78 |
| S-T22 | Female | 51 | Ⅱ | 10.28 |
| S-T23 | Female | 62 | Ⅳ | 12.06 |
| S-T24 | Female | 58 | Ⅳ | 12.21 |
| S-T25 | Female | 59 | Ⅳ | 12.59 |
| S-T26 | Female | 51 | Ⅲ | 12.81 |
| S-T27 | Female | 71 | Ⅳ | 13.07 |
| S-T28 | Female | 54 | Ⅳ | 13.56 |
| S-T29 | Female | 37 | Ⅰ | 14.17 |
| S-T30 | Female | 44 | Ⅳ | 14.39 |
| S-T31 | Female | 74 | Ⅰ | 14.56 |
| S-T32 | Female | 55 | Ⅳ | 15.15 |
| S-T33 | Female | 64 | Ⅳ | 16.91 |
| S-T34 | Female | 38 | Ⅰ | 17.09 |
| S-T35 | Female | 54 | Ⅳ | 18.49 |
| S-T36 | Female | 51 | Ⅳ | 19.35 |
| S-T37 | Female | 52 | Ⅰ | 21.20 |
| S-T38 | Female | 62 | Ⅳ | 21.87 |
| S-T39 | Female | 57 | Ⅳ | 23.18 |
| S-T40 | Female | 67 | Ⅳ | 23.57 |
| S-T41 | Female | 75 | Ⅳ | 27.16 |
| S-T42 | Female | 64 | Ⅳ | 27.93 |
| S-T43 | Female | 56 | Ⅳ | 28.86 |
| S-T44 | Female | 52 | Ⅳ | 31.45 |
| S-T45 | Female | 51 | Ⅳ | 43.89 |
| S-T46 | Female | 50 | Ⅳ | 49.97 |
| S-T47 | Female | 58 | Ⅳ | 63.32 |
| S-T48 | Female | 61 | Ⅳ | 66.94 |
| S-N1 | Female |  |  | 8.79 |
| S-N2 | Female |  |  | 8.36 |
| S-N3 | Female |  |  | 6.56 |
| S-N4 | Female |  |  | 5.09 |
| S-N5 | Female |  |  | 4.82 |
| S-N6 | Female |  |  | 4.30 |
| S-N7 | Female |  |  | 4.15 |
| S-N8 | Female |  |  | 3.38 |
| S-N9 | Female |  |  | 3.11 |
| S-N10 | Female |  |  | 3.06 |
| S-N11 | Female |  |  | 2.51 |
| S-N12 | Female |  |  | 2.33 |
| S-N13 | Female |  |  | 1.88 |
| S-N14 | Female |  |  | 1.85 |
| S-N15 | Female |  |  | 1.80 |
| S-N16 | Female |  |  | 1.39 |
| S-N17 | Female |  |  | 1.39 |
| S-N18 | Female |  |  | 1.28 |
| S-N19 | Female |  |  | 1.18 |
| S-N20 | Female |  |  | 1.14 |
| S-N21 | Female |  |  | 1.05 |
| S-N22 | Female |  |  | 1.05 |
| S-N23 | Female |  |  | 0.82 |
| S-N24 | Female |  |  | 0.78 |
| S-N25 | Female |  |  | 0.72 |
| S-N26 | Female |  |  | 0.54 |
| S-N27 | Female |  |  | 0.00 |
| S-N28 | Female |  |  | 0.00 |
| S-N29 | Female |  |  | 0.00 |

Abbreviations: PMR, percent of methylated reference.

Table S3. Primers and probes used in MethyLight assay

| Primers | Sequence |
| --- | --- |
| Forward primer for ZNF154 | GGGATTTATGAAAATTATATTATTTAGAATGTT |
| Fluorescent probe for ZNF154 | FAM-CGTTGAACGTTACGTTA-MGB |
| Reverse primer for ZNF154 | CCCGTCCTTTCTTTTTATAACTCT |
| Forward primer for ALU | GGTTAGGTATAGTGGTTTATATTTGTAATTTTAGTA |
| Fluorescent probe for ALU | VIC-CCTACCTTAACCTCCC-MGB |
| Reverse primer for ALU | ATTAACTAAACTAATCTTAAACTCCTAACCTCA |

Table S4. The sequences of sgRNAs and shRNAs

| Name | Sense Strand | Antisense Strand |
| --- | --- | --- |
| shKAP1 | CTGAGACCAAACCTGTGCTTACTC  GAGTAAGCACAGGTTTGGTCTCAG | CTGAGACCAAACCTGTGCTTACTCGA  GTAAGCACAGGTTTGGTCTCAG |
| sgRNA1 | aaacCTTGGAGTAAAAGCGAAGCT | caccgAGCTTCGCTTTTACTCCAAG |
| sgRNA2 | aaacGACCCTTTGCACCAACCTCT | caccgAGAGGTTGGTGCAAAGGGTC |
| sgRNA3 | aaacGGGACCCTTTGCACCAACCT | caccgAGGTTGGTGCAAAGGGTCCC |
| sgRNA4 | aaacTGTAGTTTTCATAGATCCCG | caccgCGGGATCTATGAAAACTACA |
| sgRNA5 | aaacCTCTTACTGGCTTAGTAGCG | caccgCGCTACTAAGCCAGTAAGAG |
| sgRNA6 | aaacTCGGTTTTCTGAGCTCTTAC | caccgGTAAGAGCTCAGAAAACCGA |
| sgRNA7 | aaacTTGTGACTCTCAAGGAAAGT | caccgACTTTCCTTGAGAGTCACAA |
| sgRNA8 | aaacTTCTTTTTGTGACTCTCAAG | caccgCTTGAGAGTCACAAAAAGAA |
| sgRNA9 | aaacCTTTCTTTTTGTGACTCTCA | caccgTGAGAGTCACAAAAAGAAAG |

Table S5. Primers for qRT-PCR

| Primers | Sequence |
| --- | --- |
| Forward primer for ZNF154 | GCTCAAAGATGCCTGTACCG |
| Reverse primer for ZNF154 | TTCCCAACTTCTCTGCAGGT |
| Forward primer for ROMO1 | ACCGTGTCAAAATGGGCTTC |
| Reverse primer for ROMO1 | ATGAATGTGCCAAAGGTGCC |
| Forward primer for KAP1 | ACCCGTCTTCAAGGTCTTCC |
| Reverse primer for KAP1 | CAGTGGCAGTAGGAGGGG |
| Forward primer for DNMT1 | ATCTTCCTGACACCCTGCAT |
| Reverse primer for DNMT1 | CCTGTCCTTCTCCCTGGTAG |
| Forward primer for DNMT3A | GGGACAAGAATGCCACCAAA |
| Reverse primer for DNMT3A | ATCCACCAAGACACAATGCG |
| Forward primer for DNMT3B | ACATGTATGATGACGATGGCT |
| Reverse primer for DNMT3B | TGCGGGAGACACATGTAACA |
| Forward primer for UHRF1 | GAGGAGGACGTCATTTACCAC |
| Reverse primer for UHRF1 | TCGGGGTTGTAGTTGAGCAT |
| Forward primer for GAPDH | GAGTCAACGGATTTGGTCGT |
| Reverse primer for GAPDH | GACAAGCTTCCCGTTCTCAG |
