# Supplementary figures and images for: Hypermethylation of ZNF154 promotes malignant potential of ovarian cancer cells by diminishing ZNF154/KAP1-mediated ROMO1 repression
Source: Cell Death Dis. 2026 May 2;17(1):582. doi: 10.1038/s41419-026-08823-w (PMC13280250; doi:10.1038/s41419-026-08823-w)

**Original Western Blot**


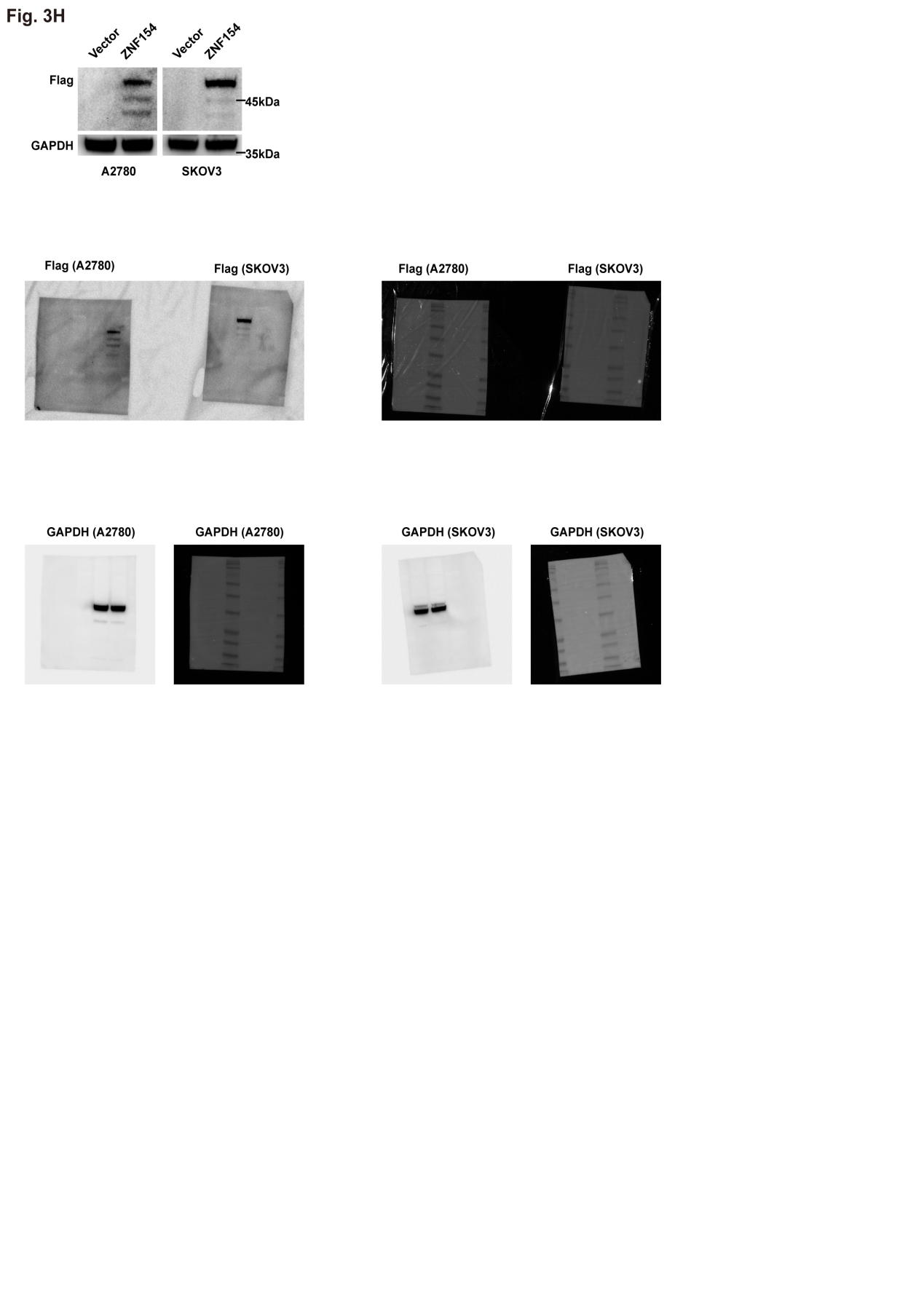


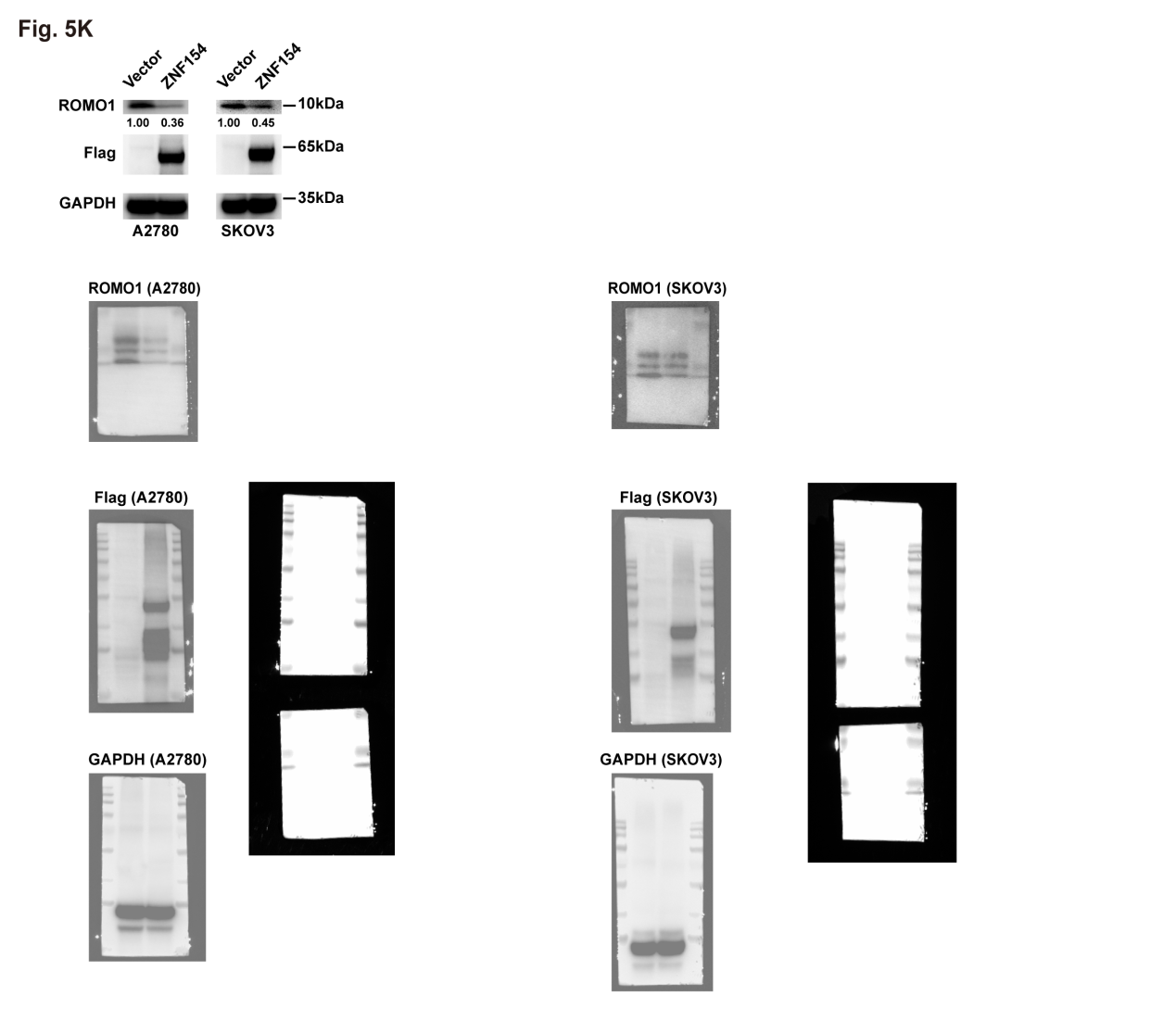


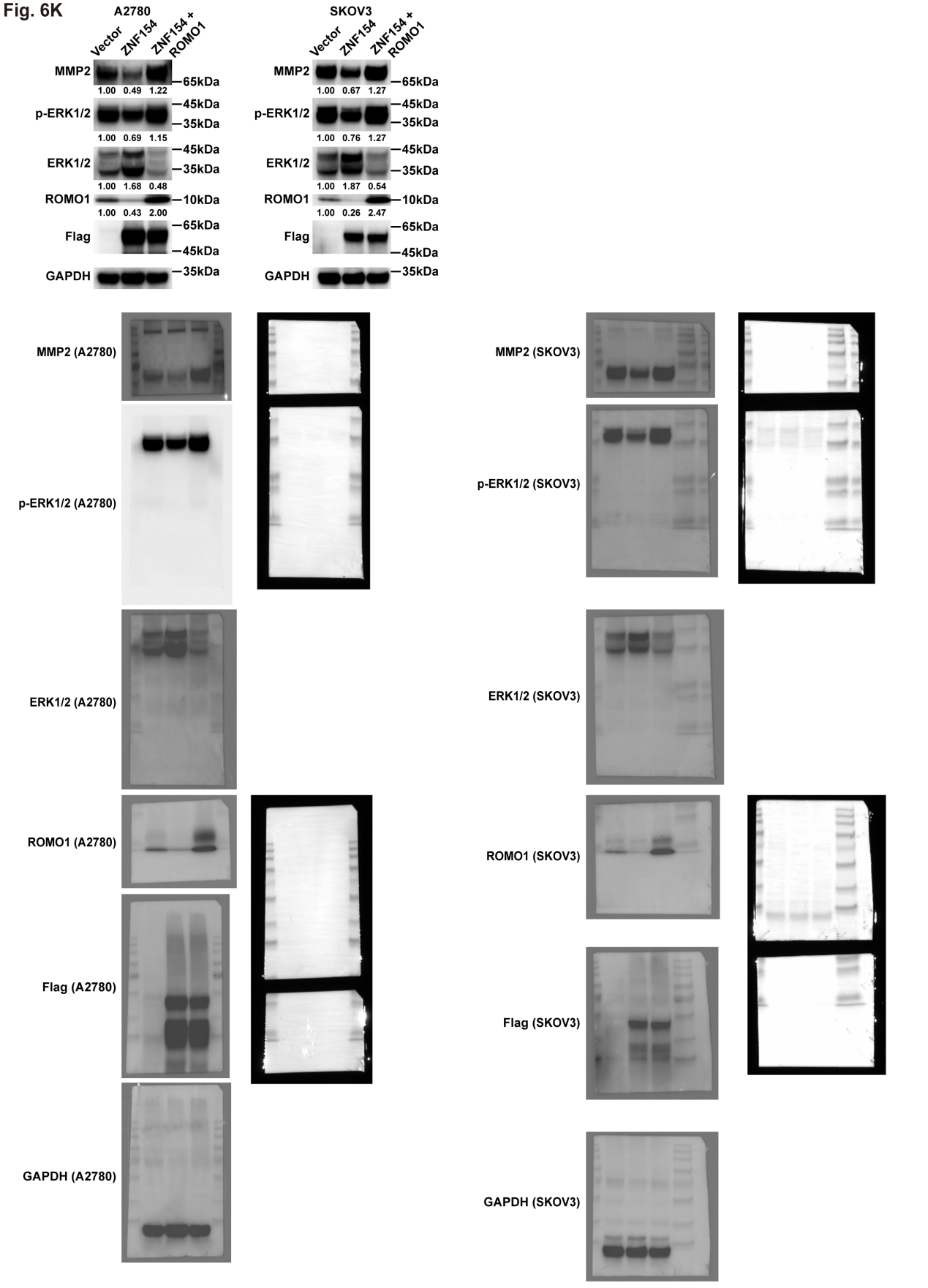


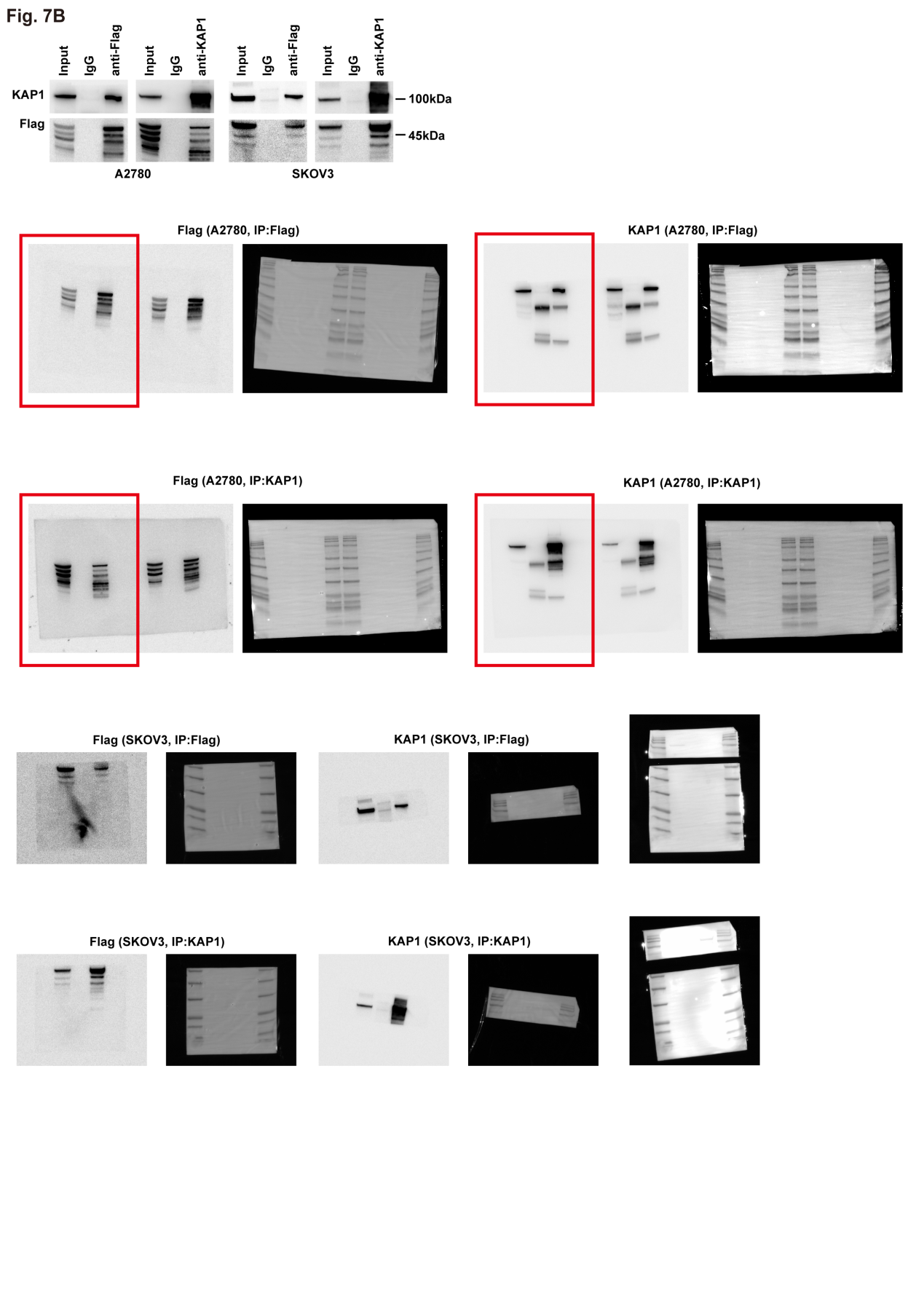


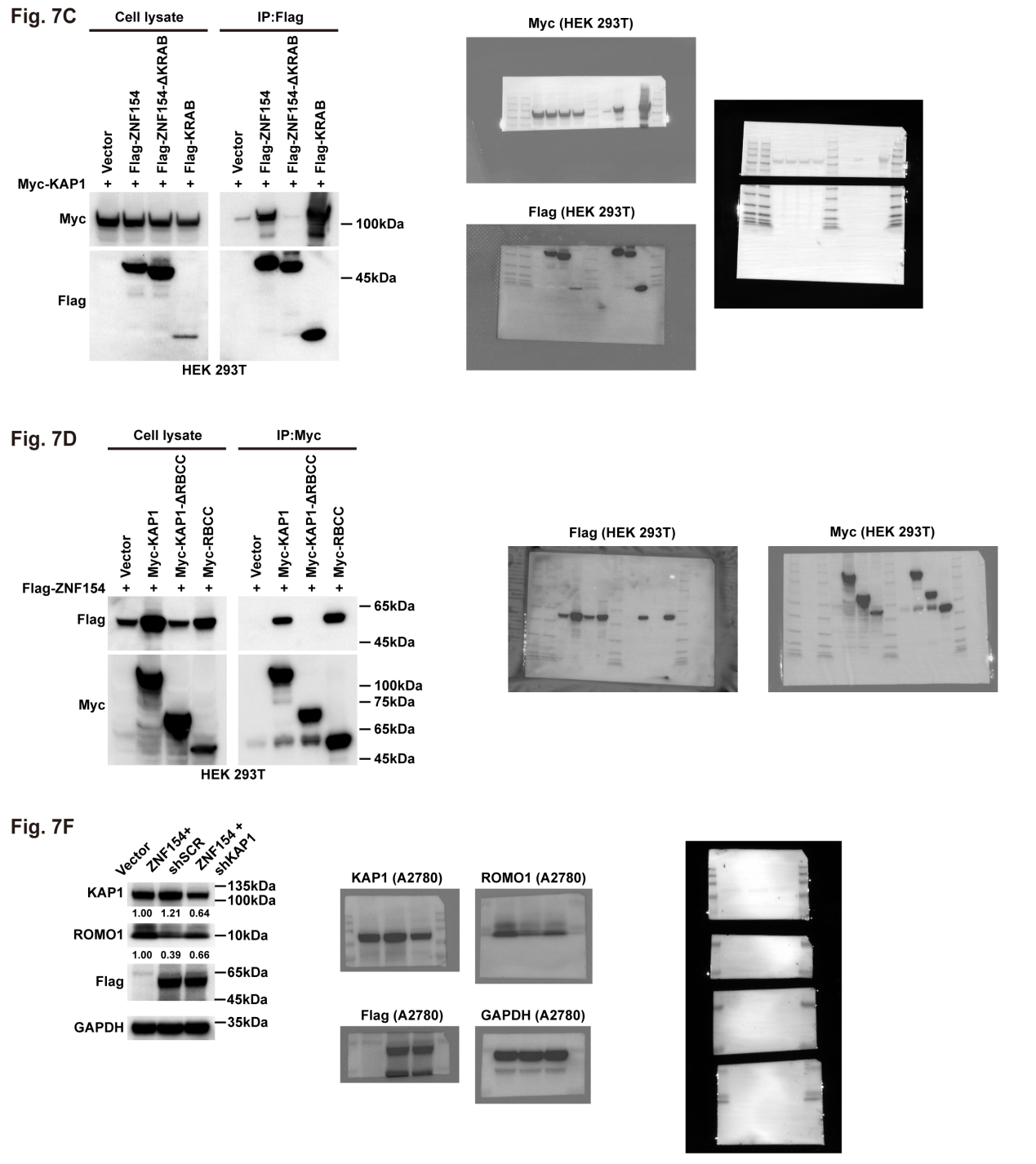


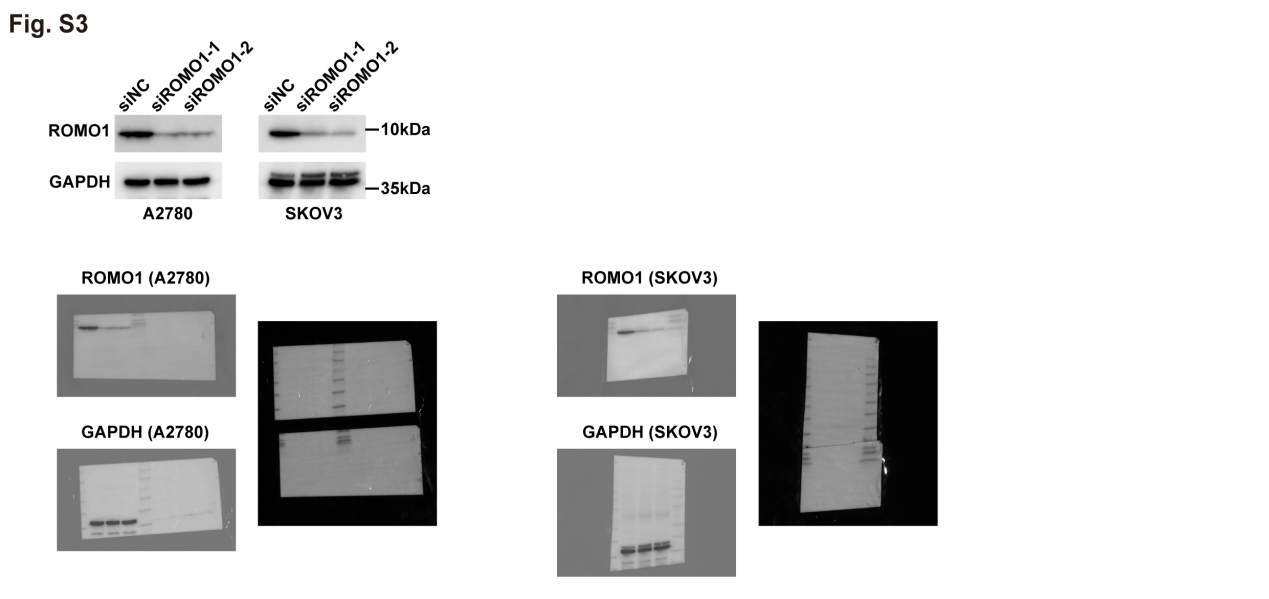

Supplement: Supplementary file 2 — Original Western Blot [file 41419_2026_8823_MOESM2_ESM.docx]
